# Biogenic synthesis of titanium nanoparticles by *Streptomyces rubrolavendulae* for sustainable management of *Icerya aegyptiaca* (Douglas)

**DOI:** 10.1038/s41598-024-81291-4

**Published:** 2025-01-09

**Authors:** Enayat M. Elqady, Eman El-said, Asmaa A. Tharwat, Lina A. Abou El-Khashab, Inas M. Y. Mostafa, Fatma Z. Hamed, Wesam M. Morsi, Mohamed M. Rezk, Inas M. Abou El-Enain

**Affiliations:** 1 Zoology and Entomology Department, Faculty of Science, Al-Azhar University (Girl Branch), Cairo, Egypt; 2 Plant Protection Research Institute, Agriculture Research Centre, Dokki, Giza Egypt; 3 Biotechnology Unit, Isotopes Department, Nuclear Materials Authority, Cairo, Egypt; 4 Botany and Microbiology Department, Faculty of Science, Al-Azhar University (Girl Branch), Cairo, Egypt

**Keywords:** Titanium oxide, Nanoparticles, *Streptomyces rubrolavendulae*, Insecticides, SDS‒PAGE, *Icerya aegyptiaca*, Biomaterials, Environmental biotechnology, Microbiology

## Abstract

Biosynthesized nanoparticles have a variety of applications, and microorganisms are considered one of the most ideal sources for the synthesis of green nanoparticles. *Icerya aegyptiaca* (Douglas) is a pest that has many generations per year and can affect 123 plant species from 49 families by absorbing sap from bark, forming honeydew, causing sooty mold, and attracting invasive ant species, leading to significant agricultural losses. The purpose of this work was to synthesize titanium dioxide nanoparticles (TiO_2_-NPs) from marine actinobacteria and evaluate their insecticidal effects on *Icerya aegyptiaca* (Hemiptera: Monophlebidae), in addition to explaining their effects on protein electrophoresis analysis of SDS‒PAGE proteins from control and treated insects after 24, 72 and 120 h of exposure. In all, seven actinobacterial isolates, the most potent of which has the potential to produce titanium hydroxide-based nanoparticles (TiO_2_-NP2), have DNA sequences that are 99.9% like those of *Streptomyces rubrolavendulae* (MCN2) according to nucleotide alignment and a phylogenetic tree. The produced TiO_2_-NPs were verified by UV examination and characterized by FT-IR, XRD, TEM, EDX, and DLS analyses. Toxicological results revealed that TiO_2_-NPs have insecticidal effects and high mortality rates reaching 55, 62.5, 80 and 95% at TiO2-NPs dose 120,250,500 and 1000 ppm respectively. Compared with the control, TiO2-NP spraying caused changes in the protein pattern of I. aegyptiaca, as indicated by the disappearance of normal bands and the appearance of other bands, as well as quantitative and qualitative changes in protein content after 24, 72 and 120 h of exposure. The application of TiO_2_-NPs by MNC2 offers a new alternative strategy to control *I. aegyptiaca* and is considered a modern approach to nanotechnology.

**Supplementary Information:**

The online version contains supplementary material available at 10.1038/s41598-024-81291-4.

## Introduction

The microbial habitat in marine ecosystems faces many deleterious effects on their survival, such as salinity gradients, temperature variations, wind dynamics, and pH fluctuations, which increase their ability to adapt to special and characteristic metabolites ^1^. Accordingly, interest in marine microorganisms is expanding in both academia and industry, suggesting that the hunting of less exploited microorganisms to gain a source of novel bioactive metabolites is valuable. In fact, unexplored habitats with unique characteristics reside in the sea ^2^. Microorganism-derived natural compounds are rich sources of medicines and therapeutic agents. The most common bacterial species in the environment is *Streptomyces*, which has the ability to manufacture a wide range of useful natural products with important biological functions in the fields of agronomy, medicine, and the food industry. However, many undiscovered natural compounds within Streptomyces exist ^3^. According to Hopwood ^4^, Streptomyces are unique among bacteria because of their linear, relatively large genomes (8–10 Mb) and unusually high G + C% (> 70%). Each genome of *Streptomyces* has many biosynthesis-related gene clusters (BGCs), which are the source of a wide variety of bioactive substances used in agriculture or medicine ^5^.

The last few decades have seen a considerable increase in interest in nanoscale materials because of their special chemical, physical, electrical, optical, and mechanical capabilities. The green synthesis of nanoparticles is highly desirable because of their easy production, low cost, uniform size and improved biomedical and environmental applications^6^. Velsankar et al.^7,8^ used a green synthesis method for the production of CuO and ZnO nanoparticles from *Panicum sumatrense* grain extracts. Recently, many researchers have directed their efforts toward demonstrating the advantages of nanoparticles as insecticides. Cu-doped ZnO-nanoparticle composites or zinc nanoparticles alone have highly devastating effects on *Spodoptera littoralis and Spodoptera frugiperda****,*** respectively***,*** under laboratory conditions^9,10^**.** Abou El-Enain et al*.*^11^ synthesized silver nanoparticles from isolated actinomycete strains and demonstrated the insecticidal effect of Ag nanoparticles on the black cutworm *Agrotis ipsilon.* Titanium dioxide (TiO_2_) is nontoxic and exhibits strong chemical and thermal stability as well as considerable resistance to corrosion. TiO_2_ is used for many different purposes, such as a photocatalyst, antibacterial agent, self-cleaning surface, solar cell, and wastewater treatment ^12^.

The Egyptian Mealybug, *Icerya aegyptiaca* (Douglas, 1890) (Hemiptera: Monophlebidae), is a polyphagous pest infesting a wide range of host plants, including ornamental plants, gardening, forestry, and many other crops ^13^. *I. aegyptiaca* has been given many common names, such as breadfruit mealybugs ^14^, and the common name that indicates that the species is native to Egypt is called the Egyptian cottony cushion scale or Egyptian fluted scale and Egyptian mealy bug ^15^. *I. aegyptiaca* can infect and feed on approximately 123 species of plants belonging to 49 plant families ^16^. This phenomenon caused a tragic flaw to the plants through the absorption of sap from the bark and the formation of honeydew, which caused the growth of sooty mold on the plants and attracted invasive ant species, resulting in agriculture annually, pollution of the plant, and the formation of fungi. Additionally, *I. aegyptiaca* causes serious cosmetic damage when its abundant white wax covers leaf surfaces and when its population density is high ^17^. With a hydrophobic wax shell, insects protect themselves from insecticides that fail to penetrate this wax shell ^18^**.** Most damage occurs when early immature scales feed on leaves, where they settle in rows along the midribs and veins and on smaller twigs. It causes decreased tree vitality, fruit drop, and defoliation ^19^. Many pesticides are used to control this pest, and although these pesticides are effective against young nymphs, they fail to affect adults ^20^. In addition, the demand for new insecticidal products has skyrocketed as insect pests have become more resistant to pesticides ^21^. To control this insect’s violent attacks, nanotechnology research is promising. It is currently considered of great importance for different sectors: industry, cosmetics, medicine, pharmaceuticals, electronics, and agriculture ^22–24^. In other industries, there is a vast array of formulations of insecticides based on nanomaterials for pest damage. As a result, nanotechnology introduces environmentally friendly, effective, and nontoxic alternatives for the control of pests in agriculture ^25^**.** In agriculture, titanium dioxide nanoparticles (TiO_2_-NPs) increase the activity of several enzymes, increase nitrate absorption, accelerate the transformation of inorganic nitrogen to organic nitrogen and increase assimilability, hence increasing vegetative growth ^26^. Protein content reflects the health status of insects, and variation in protein content is considered an important biochemical indicator of increased protein synthesis ^27^.

Therefore, the present study aims to examine marine actinobacterial isolates for titanium nanoparticle production and then investigate the effects of titanium oxide nanoparticles on the quantitative and qualitative protein profile of *Icerya aegyptiaca* after 24, 72, and 120 h of exposure to the median lethal concentration (LC_50_) of titanium oxide nanoparticles as a new alternative strategy to control *Icerya aegyptiaca*.

## Materials and methods

### Isolation of Actinomyces

Sediment samples were collected from the northern coast of the Mediterranean Sea, Marsa Matruh, Egypt, transported in a sterile container to the laboratory, and maintained at -4°C. Prior to pretreatment, all the samples were enriched for spore-forming actinomycetes via the dispersion and differential centrifugation (DDC) technique ^28^. The Actinomycete isolate was extracted from the sea sediment sample via a serial dilution technique. The media employed for isolation included starch‒nitrate agar, inorganic‒trace salt‒starch agar, and yeast extract‒ malt extract agar. Each dilution was streaked on two plates and incubated at 28 °C for seven days. The development of the actinomycetes on the plates was periodically observed. The colonies that developed were selected and cleaned on the basis of their convexity, hardness, color, and dryness ^29^. The pure colonies were chosen, subcultured, cleaned, and kept on starch nitrate agar slants at 4 °C for further study.

### Biosynthesis of TiO_2_ nanoparticles using actinomycete isolates

The ability of the Actinomyces isolates to synthesize titanium nanoparticles was examined in all the isolates. The isolates were cultured in broth media supplemented with starch nitrate. Prior to sterilization, the pH of the media was adjusted to a range of 7–8.4, and the media was incubated for seven days under submerged conditions. It was believed that this culture was the first one. After 25 mL of the original culture was diluted four times, 75 mL of starch nitrate broth medium was added. For a further week, this diluted culture mixture was left to develop. After 20 mL of 0.0025 M [Ti(OH)_2_] titanium tetra isopropoxide was added to the broth culture, which contained the actinomycete metabolites for oxidation‒reduction process propagation, the mixture was heated to 60°C in a steam bath for approximately 20 min. At that point, white deposits began to develop on the bottom of the flask, indicating the beginning of the transformation. Room temperature was used for the incubation of the actinomycete isolate-containing broth culture. After 12 to 48 h (h), the broth culture was checked to determine whether a noticeable coalescent white cluster had formed at the bottom, verifying TiO_2_-NP production ^30^.

### Identification of actinomycete-producing TiO_2_ nanoparticles

The cultural and morphological characteristics of the actinomycete isolate MNC2 were determined via International Streptomyces Project (ISP) media and scanning electron microscopy (FESEM, QUARTO S, Thermo Fisher, USA) at the Nano Technology Regional Centre (BUE) ^31^. For molecular identification of the actinomycete isolate and phylogenetic studies, the actinomycete isolate was grown on a starch agar slant for 7 days. Two millilitres of suspension was inoculated into starch nitrate broth and incubated in a shaking incubator for 14 days at 200 rpm and 30°C. Genetic material from cultured microorganisms was purified via a polymerase chain reaction (PCR) product extraction kit. (Qiagen, Valencia). DNA sequences were obtained via an Applied Biosystems 3130 genetic analyser (HITACHI, Japan), and a BLAST analysis (Basic Local Alignment Search Tool) was initially performed to establish sequence identity to the GenBank accessions. The phylogenetic tree was created with Meg. The alignment module of the Laser gene Deoxyribonucleic acid (DNA) Star version 12.1 Phylogenetic analyses were performed via maximum likelihood, neighbour-joining, and maximum parsimony in MEGA6^32,33^. Molecular analysis of the isolate in this study was performed at the Biotechnology Unit of Cairo University Research Park (CURP), Reference laboratory for veterinary quality control on poultry production, (physiology Department) Plant Protection Research Institute, Dokki, Giza, Egypt.

### Characterization of the synthesized TiO_2_ nanoparticles

The synthesized TiO_2_ nanoparticles were characterized by ultraviolet–visible (UV–vis), Fourier transform infrared (FTIR), X-ray diffraction (XRD), scanning electron microscopy (SEM), energy-dispersive X-ray spectroscopy (EDX), and zeta potential analyses. The functional groups present in the synthesized TiO_2_ nanoparticles were confirmed by FTIR via an Agilent Cary 630 FTIR spectrometer. The average particle size and morphology of the synthesized TiO_2_ nanoparticles were determined via SEM by an Evo 15 Zeiss SEM, UK. The elemental composition of the synthesized TiO_2_ nanoparticles was identified via EDX by an Evo 15 Zeiss SEM, UK-. XRD of the synthesized TiO_2_ nanoparticles was performed via Panalytical Xper PRO. The particle size and size distribution of the synthesized TiO_2_ nanoparticles were determined via DLS via a Zetasizer Ver. 6.32, MAL1071664. UV–vis analysis of the synthesized TiO_2_ nanoparticles was performed (GBC, Contrary to 3030).

### Toxicity bioassay procedure

The bioassay studies were conducted according to the procedure described by EL-Hefny, et al. ^34^ with some modifications.

### Direct spray method

In this study, *Ficus nitida* shrubs subjected to heavy infestations of *I. aegyptiaca* were chosen, and the plants did not receive any insecticide treatment. Samples of infested *Ficus* leaves were collected randomly from different infested shrubs (each consisting of 8 leaves per treatment), kept in paper bags, and transferred to the laboratory. Treated leaves were kept in glass Petri dishes (6 cm in diameter), and in each Petri dish, tissue paper was placed for dehydration of the acceded fluid. Twenty adult females of *I. aegyptiaca* were found in each replicate at the tested concentrations (125, 250, 500, and 1000 ppm). Each treatment was replicated three times for each concentration (3 replicates/conc).

A particular concentration of each tested biosynthesis TiO_2_-NP was sprayed directly on *I. aegyptiaca* adult females via a hand sprayer (20 mL), and the Petri dish was covered with a nylon net. The sprayed water was distilled water as a negative control, and the recommended insecticide (malathion) was used as a positive control. Cotton mealy bug mortality was determined at 24, 72, and 120 h following the initial application. The mortality records for all the treatments were obtained as percentages.

### Protein analysis

Before protein extraction, the samples from the control and treated insect groups were washed with phosphate buffer solution (pH 7.2). The total protein of each group was assayed on ice via the addition of 1 mL of lysis buffer (including 10% glycerin, 2.5% SDS, 5% β-mercaptoethanol, and 62.5 mM Tris–HCl, pH 6.8) per mg of sample. The extraction mixture was subsequently left for 10 min at room temperature, after which the samples were sonicated in an ice bath. The samples were subjected to centrifugation at 20,000 × g, and the supernatant was aliquoted and stored at -20 °C. The protein concentration was measured according to the method described by Bradford ^35^.

### SDS‒PAGE analysis

SDS–PAGE was performed according to the methods of Laemmli ^36^. Before the samples were subjected to SDS‒PAGE, they were dissolved (100 mg/sample). Then, S–PAGE loading buffer (1 sample : 4 sample buffer were mixed (10% SDS, 20% Glycerol, 0.2 M tris pH 6.8, 10 mM beta-mercapto-ethanol and 0.05% bromophenol blue) and were heated on 95 °C for 5 min, centrifuged at 20,000 × g for 10 min, loaded on a 4% stacking gel (containing 6.1 mL dH_2_O, 1.3 mL (29.2% acrylamide and 0.8% bis-acrylamide), 2.5 ml 0.5 M tris pH 6.8, 100 µl 10% SDS, 100 µl 10%APS and 100 µl TEMED) and 15% separating gel (2.4 mL distilled water (dH_2_O), 5 mL (29.2% acrylamide and 0.8% bis-acrylamide), 2.5 mL 1.5 M tris pH 8.8, 100 µl 10% SDS, 100 µl 10%APS and 100 µl TEMED). Then, running buffer was prepared (25 mM Tris–HCl, 200 mM glycine and 0.1% (W/V) SDS). The run started (80 V for 4 h). After that, the gel was stained in staining solution (50% dH_2_O, 40% methanol, 10% glacial acetic acid and 0.1% Coomassie brilliant blue) for 20 min with gentle agitation. Then, the mixture was destained overnight in destaining solution (50% dH_2_O, 40% methanol and 10% glacial acetic acid). The gel was photographed and scanned. The molecular weight and relative fragmentation (Rf) of the proteins in the profiles of the control and treated insect samples were determined and compared with those of protein markers (from 245–11 kDa).

### Statistical analysis

The means were compared via Tukey’s honestly significant difference (HSD) test at p ≤ 0.05 when the ANOVA results were significant and were analysed via the Statistics program (version 9.0). The lethal concentration value LC and lethal time LT ^37^ with 95% confidence limits were calculated via regression equations (Y = b*x + a), and regression coefficients (R^2^) were calculated via the SPSS program (version 20.0). The graphs were drawn with GraphPad Prism (version 9.5.1).

## Results

### Isolation of Actinomyces and biosynthesis of TiO2 nanoparticles

Seven Actinomycete isolates were separated from samples of sea sand and tested for their ability to produce TiO_2_ nanoparticles. In the present study, TiO_2_-NPs were created and observed by the naked eye as a coalescent white cluster.

### Identification of actinomycete-producing TiO_2_ nanoparticles

**Fig. 1:**
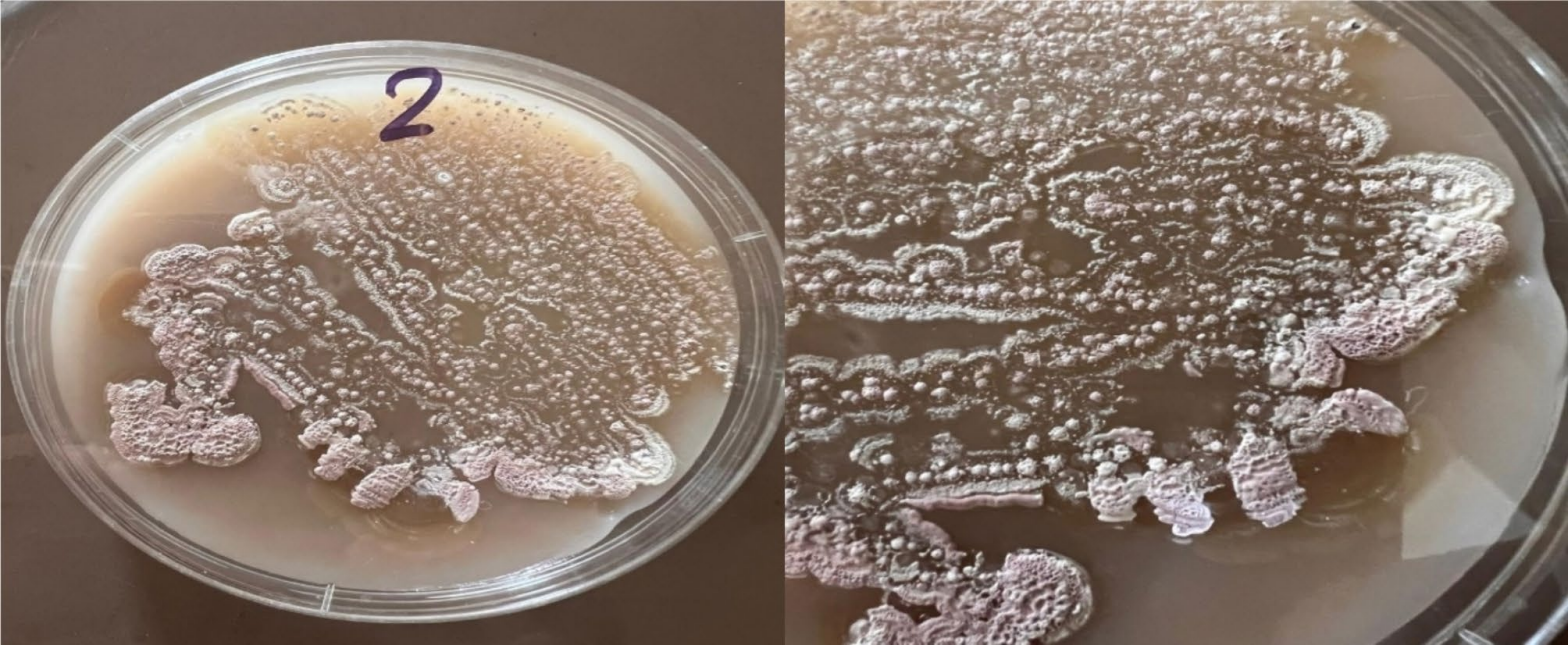
Actinomycete growth in petri dishes on starch nitrate agar media.

**Fig. 2:**
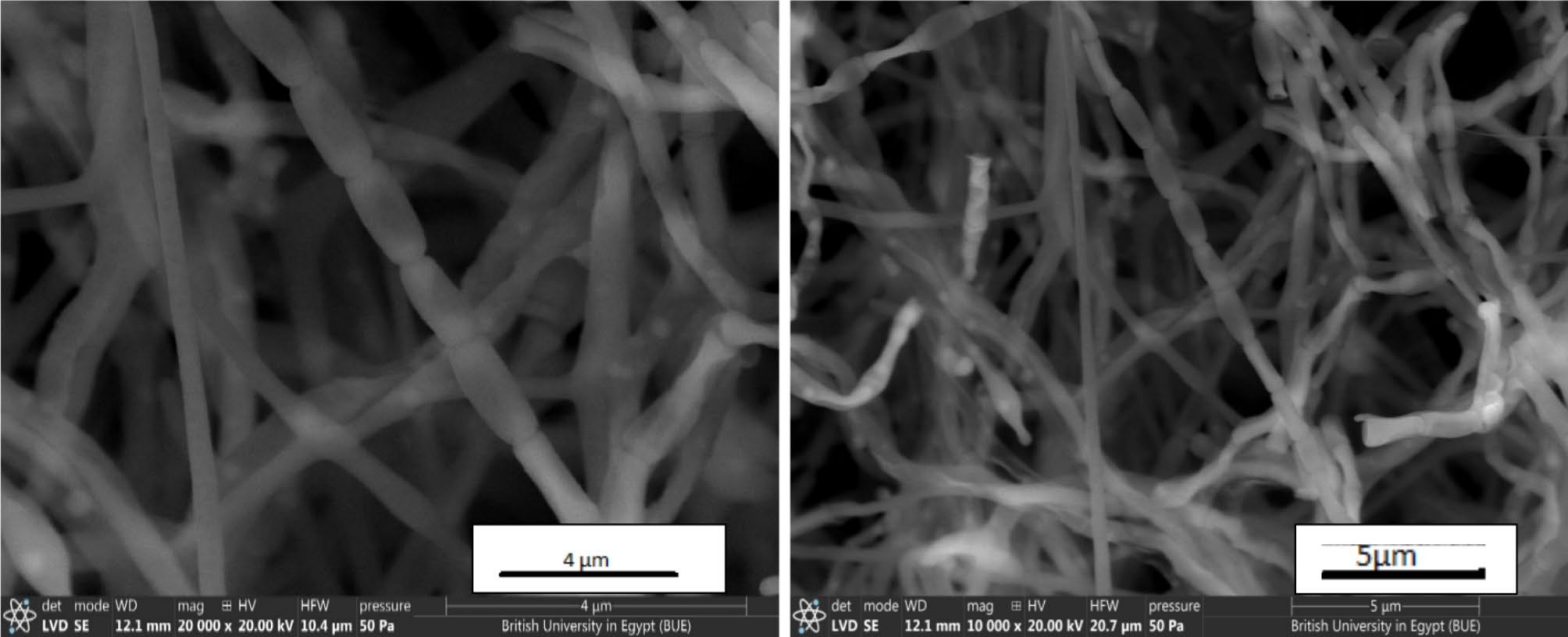
Scanning electron microscopy of Streptomyces isolate MNC2 (20,000 X).

The culture and morphological characteristics of the actinomycete isolates grown on different ISP media are tabulated in Tables 1 and 2. The spore mass of the isolate was pinkish gray, but the reverse was yellow brown, no diffusible pigments were formed, and melanin pigments were formed. Figure 1 shows the growth of the actinomycete in the Petri dish, and the morphological traits of the organism are displayed via electron micrographs and ocular examinations of the isolate. As shown in Fig. 2, the spore chain was made up of *Rectiflexible* spores with smooth surfaces.

**Table 1:** Cultural characteristics of the actinomycete isolate MNC2 growing on ISP media.

Type of media	Growth	Colour of spore mass	Colour of the reverse	Colour of diffusible pigments
Inorganic-trace salt- starch agar	Moderate	Pinkish grey	Yellow brown	None
Glycerol asparagine agar	Good	Pinkish grey	Yellow brown	None
Yeast extract- malt extract agar	Weak	white	yellow	None
Oatmeal agar	Weak	White	Yellow	None
Tryptone yeast extract broth	Weak	White	Pale yellow	None
Peptone yeast extract iron agar	Good	Pinkish grey	Yellow brown	None

**Table 2:** Morphological characteristics of the actinomycete isolate MNC2.

Morphological characteristics	Spore chain	Spore surface	Spore mass colour	Motility	Colour of substrate mycelium	Diffusible pigment	Melanin formation
Results	*Rectiflexibiles*	Smooth	Pinkish grey	Non motile	Yellow brown	None	Formed

**Fig. 3:**
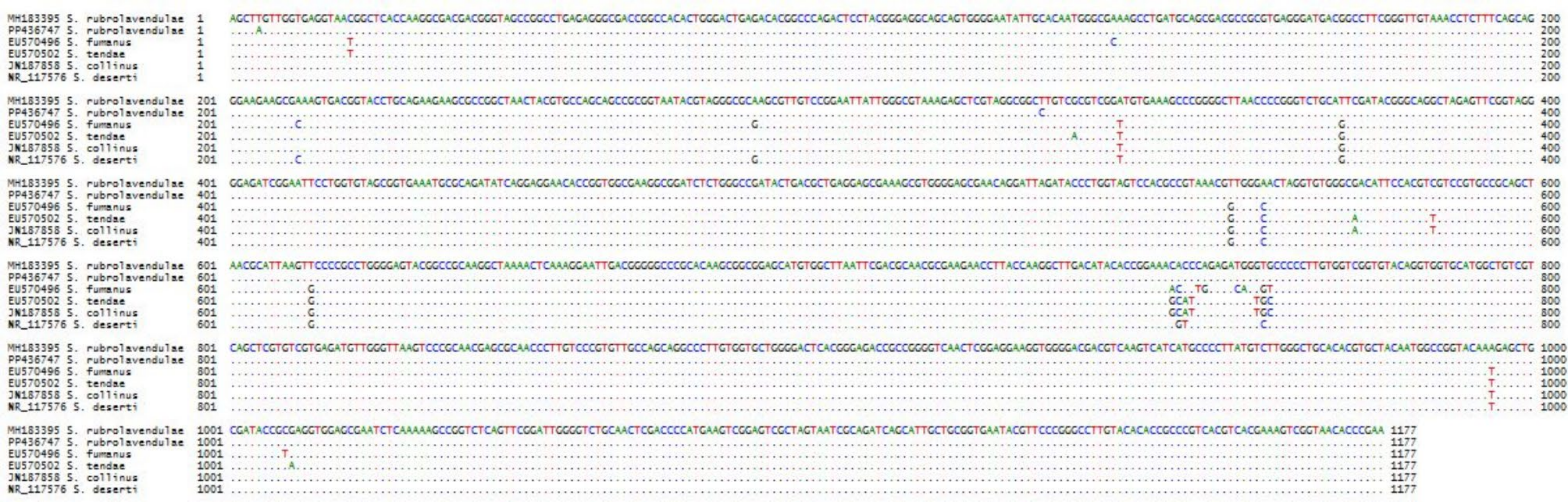
Nucleotide alignment of the actinomycete isolate MNC2**.**

**Fig. 4:**
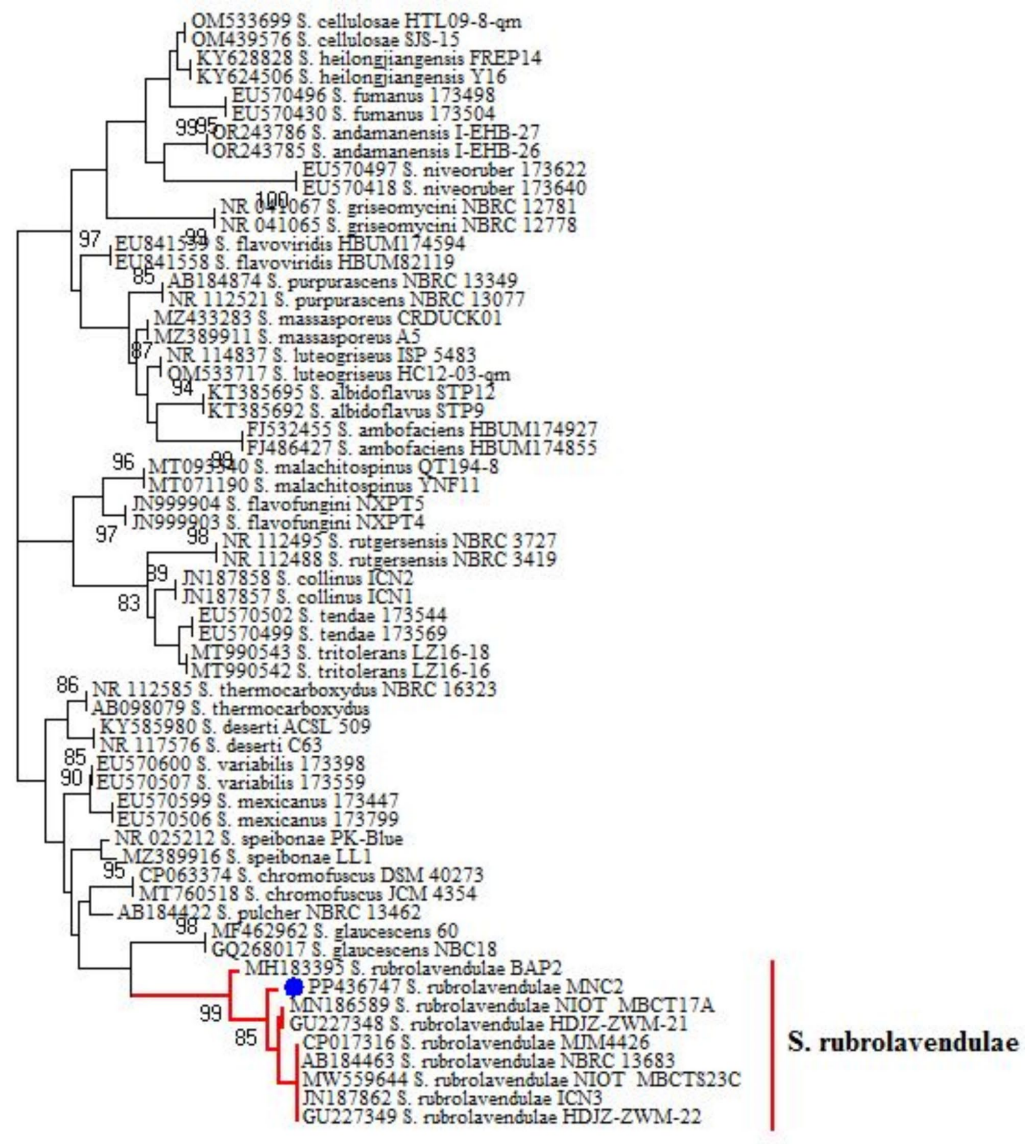
Phylogenetic analysis of Streptomyces isolate MNC2 via neighbor joining.

The actinomycete isolate was identified with the aid of Streptomyces sp. and the 16S rRNA sequences of the chosen MNC2 isolate, which were identified via multiple sequence alignment. The results of PCR amplification were investigated via agarose gel electrophoresis. This isolate is 99.9% like *Streptomyces rubrolavendulae*, according to the nucleotide alignment in Fig. 3 and the phylogenetic tree in Fig. 4. The authors submitted the sequence to the GenBank1 Nucleotide Sequence Database and the following GenBank accession number(s) for your nucleotide sequence(s): SUB14289766 Streptomyces PP436747. https://www.ncbi.nlm.nih.gov/nuccore/PP436747.

### FTIR analysis

**Fig. 5:**
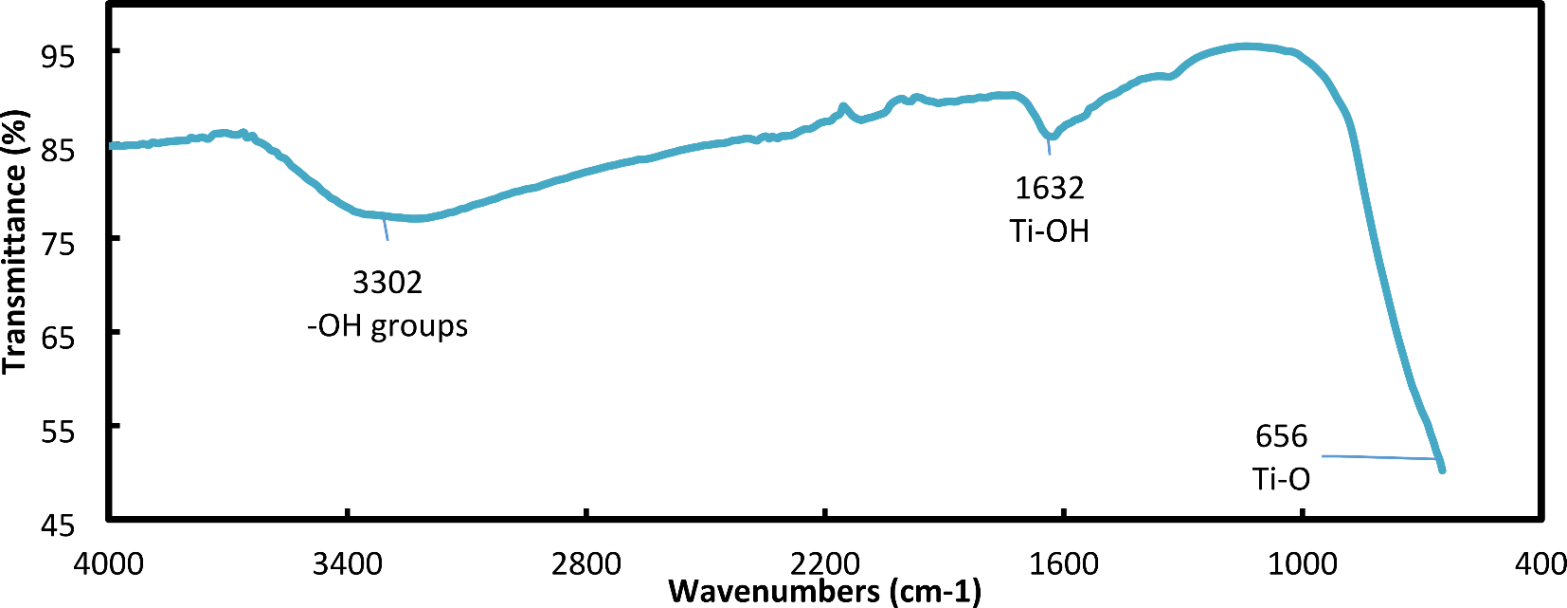
FTIR spectrum of the synthesized TiO_2_ nanoparticles.

FTIR analysis (Fig. 5) is an effective analytical technique used to detect the functional groups present in materials to elucidate their composition. The FTIR spectrum of titanium dioxide (TiO_2_) nanoparticles contain characteristic absorption bands associated with surface OH (broad band at 3302 cm^-1^), Ti–OH bonds (1632 cm^-1^) and Ti–O bonds (656 cm^-1^). This suggests that the synthesized nanoparticles were TiO_2_ anatase in structure.

### TEM, EDX and zeta potential analysis

**Fig. 6:**
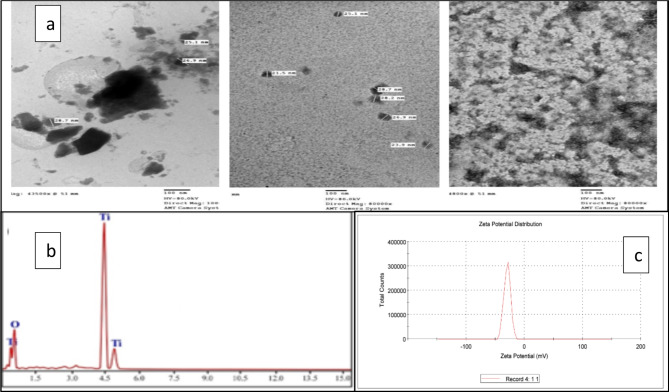
**(a)** TEM image of the synthesized TiO_2_ nanoparticles. **(b)** EDX analysis. **(c)** Zeta potential.

As illustrated in Fig. 6, the morphology of the synthesized TiO_2_ nanoparticles is shown in the TEM image as an agglomerate of spherical TiO_2_ nanoparticle beads. The agglomeration of synthesized TiO_2_ nanoparticles may be due to the adhesion of particles to each other by physical forces. Additionally, the nano size and shape of the synthesized TiO_2_ nanoparticles were confirmed by average grain sizes ranging from 21.5–28.7 nano-meters and spherical shapes (Fig. 6a). According to EDX analysis (EDX, Fig. 6a), the synthesized TiO_2_ nanoparticles are composed of oxygen (47.52 weight % and 73.04 atomic %) and titanium (52.48 weight % and 26.96 atomic %), as shown in Table 3. Additionally, zeta potential tests revealed that TiO2-NPs carried negative charges on their surface (Fig. 6b and 6c).

**Table 3:** EDX analysis (b) of the synthesized TiO_2_ nanoparticles.

Element	Weight %	Atomic %	Net Int	Error %	R	A	F
O K	47.52	73.04	1036.60	10.81	0.8800	0.0429	1.0000
Ti K	52.48	26.96	8541.50	1.73	0.9348	0.9459	1.0152

### XRD analysis

**Fig. 7:**
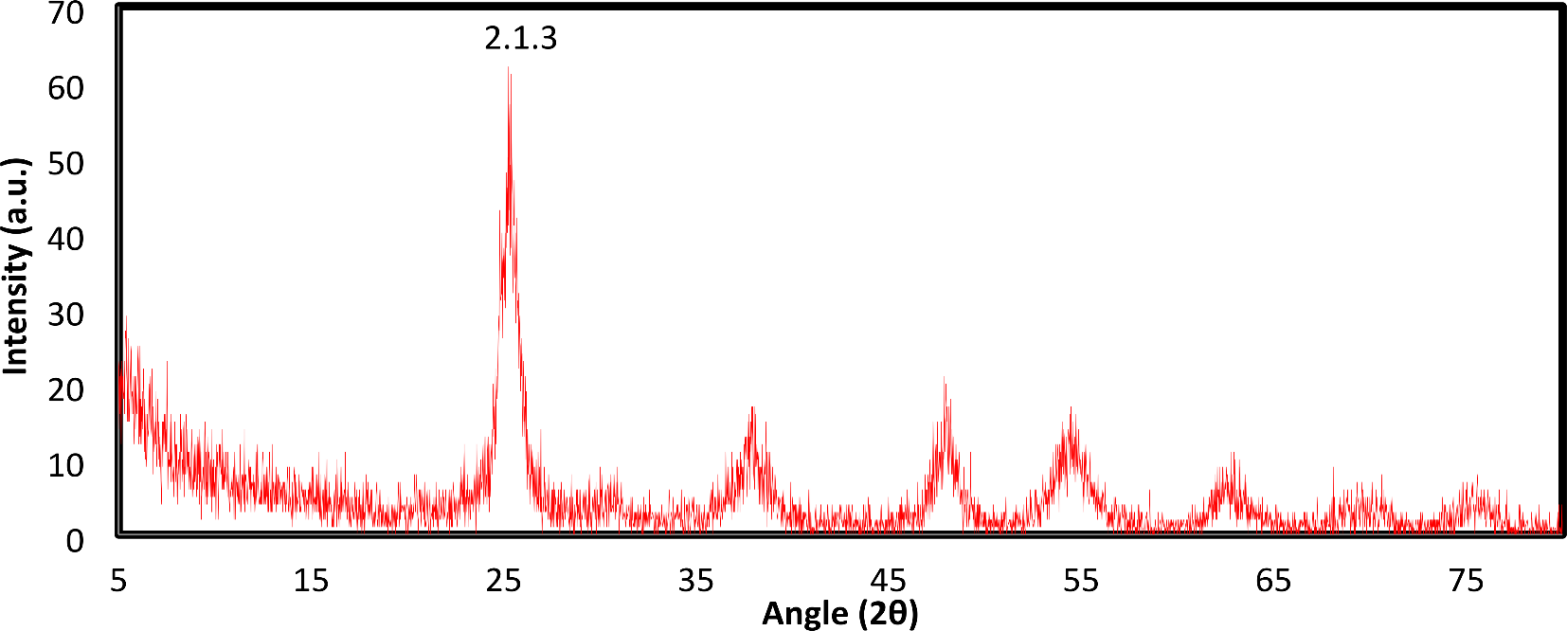
XRD pattern of the synthesized TiO_2_ nanoparticles.

XRD analysis is an effective analytical technique for analysing and determining the crystal size and structure of crystalline materials. Generally, titanium dioxide crystallizes into three structures, i.e., rutile (tetragonal), brookite (orthorhombic), and anatase (tetragonal). Figure 7 shows the XRD pattern of the synthesized TiO_2_-NPs. The diffraction peaks were observed at 2θ values of 10.3, 15.3, 16.7, 25.4, 30.8, 37.9, 47.9, 54.5, 58.5, and 77.8. These peaks are in good agreement with the characteristic peaks of TiO_2_ nanoparticles (Ref. Code: 01–075-2547). The diffraction peaks revealed that the formed nanoparticles were in the anatase and crystalline phases **(**Fig. 7**).**

### UV‒visible spectroscopy

**Fig. 8:**
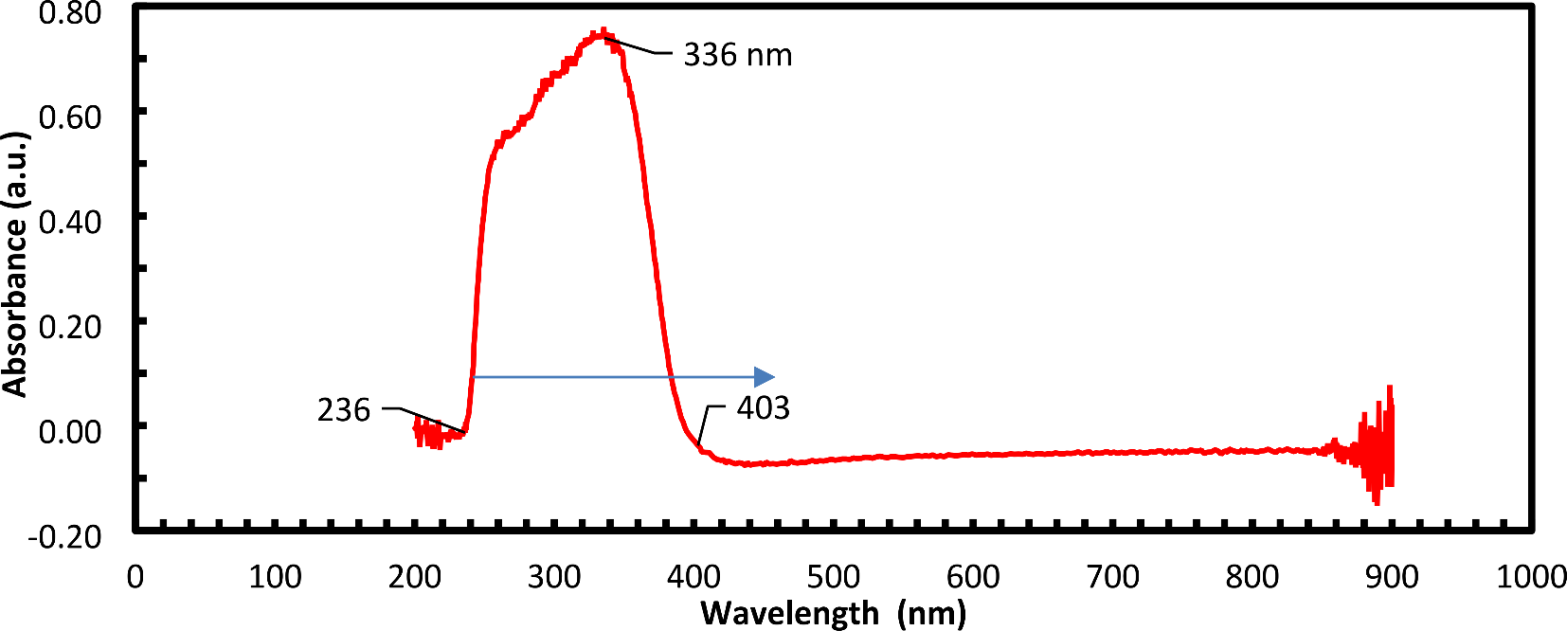
UV‒visible spectrum of TiO_2_ nanoparticles.

The UV–Vis absorbance spectra of the produced TiO_2_ nanoparticles (200–800 nm) revealed a good absorption band in the 236–403 nm region (Fig. 8). Therefore, the nanoparticles absorb light from the visible and UV regions at 236–403 nm, which may be useful in their visible and UV light response applications.

### Toxicological bioassay

**Fig. 9:**
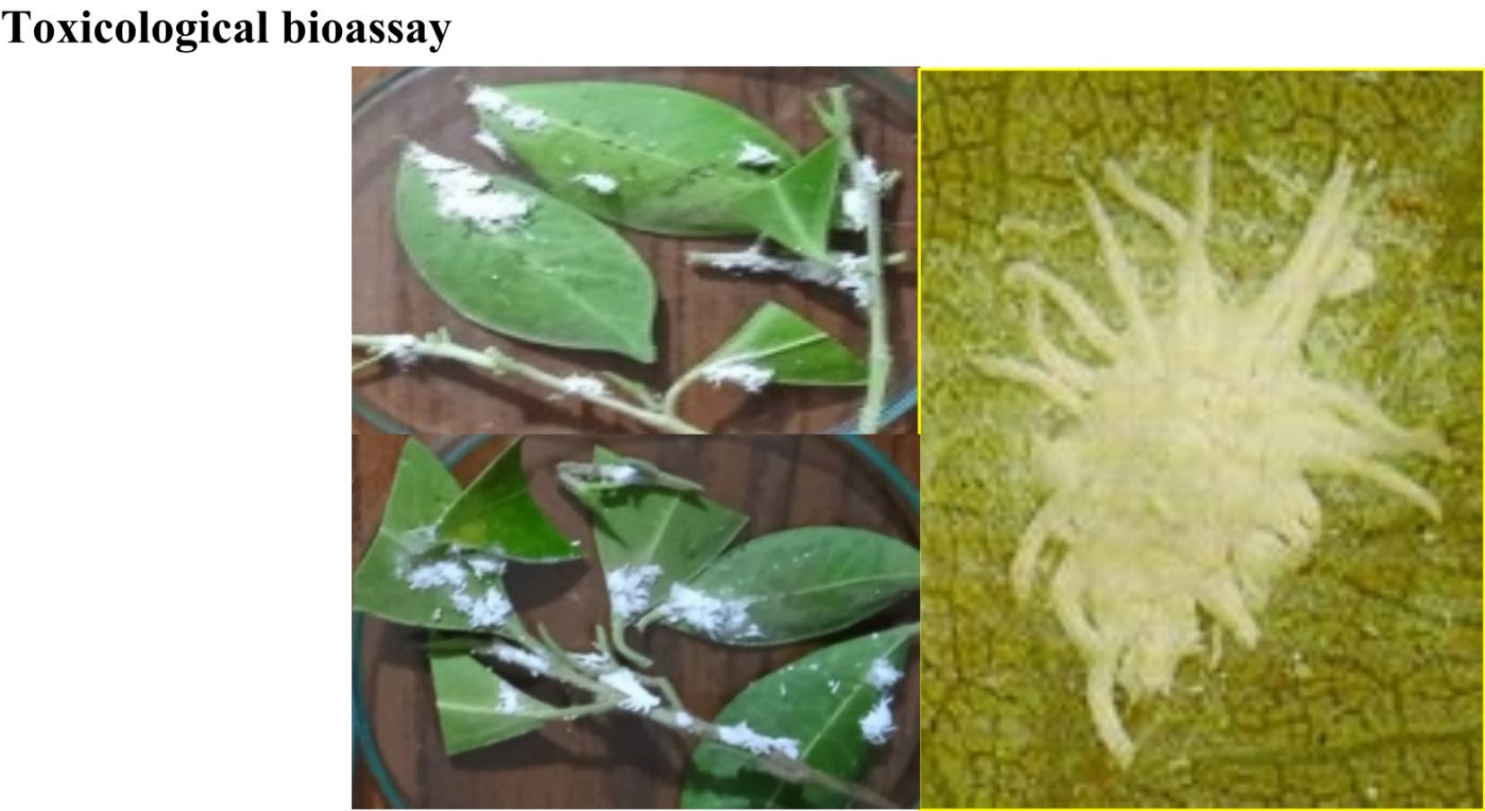
shows the *Icerya aegyptiaca* adult.

The *Icerya aegyptiaca* adult appeared as an elongated oval broad shape, covered by white wax and seemed to orange appearance (Fig. 9).

### Insecticidal effects of the Bio-TiO_2_-NPs on ***Icerya aegyptiaca*** adult

**Fig. 10:**
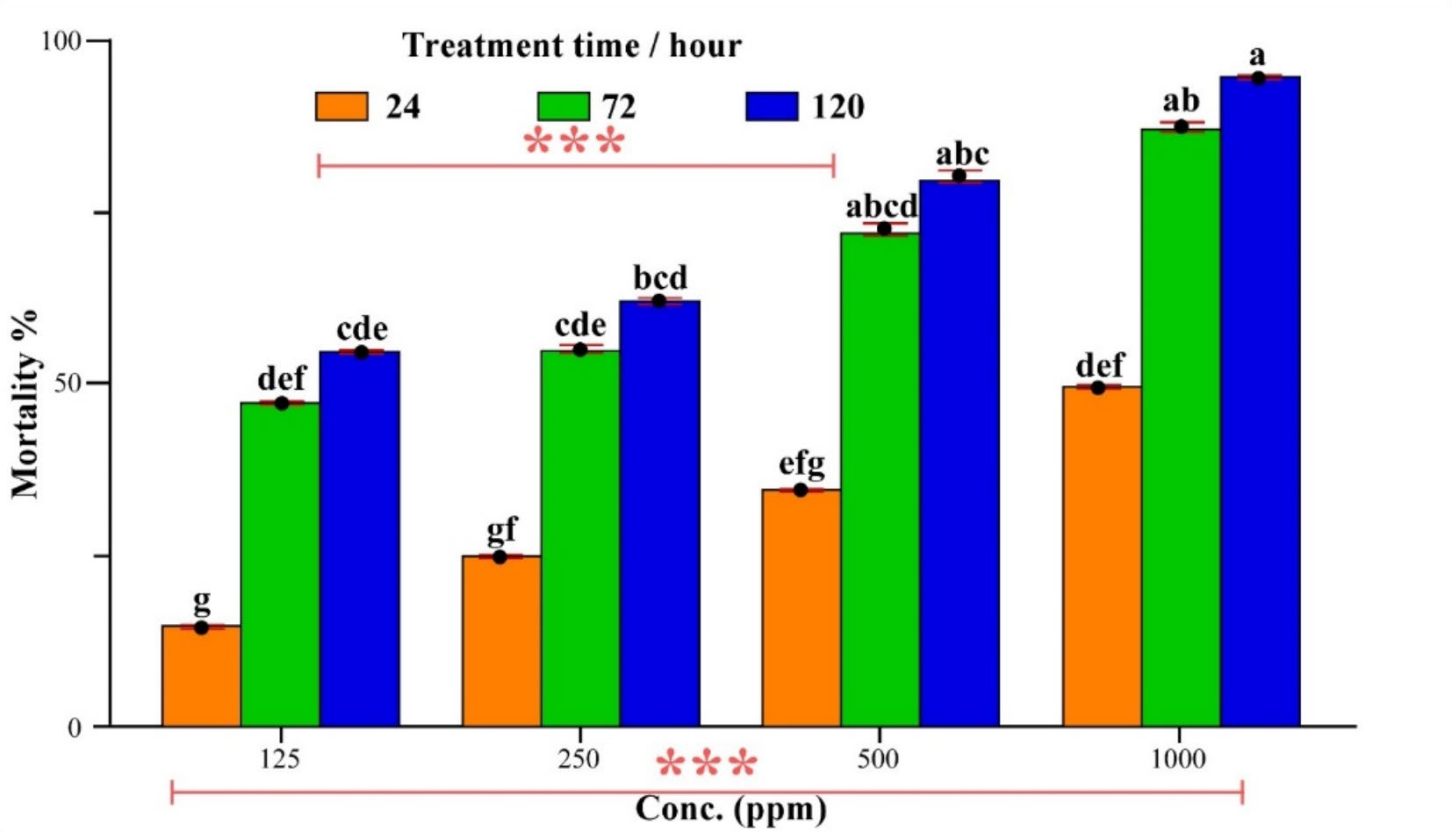
Mortality percentage of *I. aegyptiaca* adult females treated with the tested biosynthesized TiO_2_-NPs. *** indicates significance at the 1% level for the treatment time and tested concentration. Different letters indicate significance according to Tukey’s HSD test at p ≤ 0.05.

The insecticidal activity of bio-TiO_2_-NPs in terms of mortality% at the tested concentrations (125, 250, 500, and 1000 ppm) after 24, 72, and 120 h compared with the recommended insecticide (malathion) as a positive control against *I. aegyptiaca* females is shown in Table 4 & Fig. 10. In terms of bio TiO_2_-NP concentrations and exposure times, analysis of variance (two-way ANOVA) revealed significantly different mortality rates in adult females at P ≤ 0.05. The mortality results revealed that the bio-TiO_2_-NPs had good insecticidal activity, with promising values at all concentrations. The lowest mortality percentage was observed at 24 h for conc. 125 ppm (15.00 ± 0.29), and the highest percentage (95.00 ± 0.29) was observed at 120 h for conc. 1000 ppm. The values of the Eta square η2 for the tested bio-TiO_2_ and positive control samples were greater than 0.14, which indicates that the impact of the tested pesticides on *I. aegyptiaca* is greater and that bio-TiO_2_ is more effective than malathion is, where η2 = 0.391 and 0.298, respectively.

**Table 4:** Mortality % of *I. aegyptiaca* adult females treated with the tested biosynthesized TiO_2_-NPs compared with malathion.

Conc/ppm	Bio-TiO_2_-NPs η2 = 0.391			Malathion* η2 = 0.298		
	Time/hour			Time/hour		
	24	72	120	24	72	120
125	15.00 ± 0.29 g	47. 50 ± 0.25def	55.00 ± 0.29cde	25.00 ± 0.65f.	60.00 ± 0.91cde	80.00 ± 0.91abcd
250	25.00 ± 0.29 fg	55.00 ± 0.65cde	62.50 ± 0.49bcde	45.00 ± 0.65 ef	75.00 ± 0.29abcde	87.50 ± 0.25abcd
500	35.00 ± 0.29efg	72.50 ± 0.85abcd	80.00 ± 0.82abc	57.5 ± 0.48 def	85.000 ± 0.65abcd	92.50 ± 0.48abc
1000	50.00 ± 0.41def	87. 50 ± 0.75ab	95.00 ± 0.29a	65.00 ± 1.32 bcde	95.00 ± 0.29ab	100 ± 0.00a
Control	00.00 ± 0.00	00.00 ± 0.00	00.00 ± 0.00	00.00 ± 0.00	00.00 ± 0.00	00.00 ± 0.00
F-statistic	for conc.(ppm) = 31.69*** for time (hour) = 73.63***			for conc.(ppm) = 12.370 *** for time (hour) = 42.105***		
P value	0.000			0.000		
*L.S.R	2.57 ± 0.74			12.370 ± 0.94		

*Positive control: Different litter indicate significant at P ≤ 0.05 according to Tukey HSD post Hoc. Test, Mortality % ± S.E., L.S.R Least Significant Range η2 Eta square for effect size ≥ 0.14.

Moreover, the probit-transformed response analysis revealed an excellent relationship between concentration and treatment time, where R^2^ = 0.997, 0.960, and 0.933 for 24, 72, and 120 h, respectively, compared with the positive control. Furthermore, the lethal concentrations with lower and upper limits at 95% = 1029.17 (728.039–1920.933) and 14,020.118 (5340.356–103,644.417) killed 50% and 90% of the insect population, respectively, after 24 h of treatment, whereas the LC_50_ = 163.583 (110.52–211.722) and LC_90_ = 1507.980 (993.476–3152.768) after 72 h of treatment. Moreover, the lethal concentrations at 120 h were 124.112 (82.022–161.662) and 834.891 (617.751–1364.969) for 50% and 90% population killing, respectively, as described in Table 5. Additionally, the results of the mortality time studies for TiO_2_-NPs are summarized in Table 6. Moreover, the lethal time values (LT _(50, 90)_ per h) of the biosynthesized TiO_2_-NPs against *I. aegyptiaca* adult females were studied, and the results revealed an increase in the concentration from 125 to 1000 ppm, which decreased the time of killing. Additionally, the LT50 values for *I. aegyptiaca* females were 91.212 (74.111–121.225), 66.600 (52.493–86.502), 37.804 (28.856–46.190), and 23.934 (17.885–29.307) at concentrations of 125, 250, 500, and 1000 ppm, respectively. On the other hand, the LT_90_ values for 125, 250, 500, and 1000 ppm were 518.677 (303.722–1432.738), 504.317 (280.938–1649.780), 191.416 (138.368–330.467), and 82.941 (67.888–110.449), respectively. According to the regression equations and regression coefficients, there are positive correlations between (lethal concentration LC and killing time LT (X independent variable) and mortality % (Y dependent variable), in addition to excellent slopes (b) for testing bio-TiO_2_ NPs [Y = 2*X + (-4.0)] and [Y = 2.5*X + (-3.25)] at concentrations of 1000 ppm and 120 h, respectively, compared with malathion, where 1*x + (-1.2) and 2.5*X + -(3) at concentrations of 1000 ppm and 120 h, respectively.

**Table 5:** Toxicity effect expressed as the lethal concentration (ppm) of the biosynthesized TiO_2_-NPs against *I. aegyptiaca* adult females compared with the recommended insecticide (malathion).

	Time/Hour	LC_50_ (Lower-Upper limits)	LC_90_ (Lower-Upper limits)	Slope ± SE	* x^2^	*R^2^	*Y =
Bio-TiO_2_-NPs	24	1029.17 (728.04- 1920.93)	14,020.118 (5340.356–103,644.417)	1.130 ± 0.205	0.092	0.997	1.2*x + (-3.6)
	72	163.583 (110.52–211.722)	1507.98 (993.476–3152.768)	1.329 ± 0.206	1.998	0.96	1.4*x + (-3.0)
	120	124.112 (82.022–161.662)	834.891 (617.751–1364.969)	1.548 ± 0.226	4.074	0.933	2*x + (-4.0)
*Malathion (P.C.)	24	392.294 (302.851- 519.271)	5088.729 (2536.931—19,678.667)	1.151 ± 0.195	1.895	0.952	1.2*x + (-3.2)
	72	86.136 (46.730- 122.006)	651.593 (487.122- 1055.793)	1.458 ± 0.237	0.465	0.987	1.5*x + (-2.7)
	120	37.087 (9.182- 67.602)	278.632 (209.348- 395.166)	1.463 ± 0.313	3.664	1.00	1*x + (-1.2)

*Significant Pearson goodness-of-fit test chi-square X^2^ ≥ 0.05 for all probit models- *R^2^ regression coefficient *Y equation regression *P.C. positive control.

**Table 6:** Lethal time (LT/ hour) dose of the biosynthesized TiO_2_-NPs against *I. aegyptiaca* adult females compared with malathion.

	Conc ppm	LT_50_ (Lower-Upper Limits)	LT_90_ (Lower-Upper limits)	Slope ± SE	*X^2^	*R^2^	*Y =
Bio-TiO_2-_NPS	125	91.212 (74.11- 121.23)	518.677 (303.722- 1432.74)	1.698 ± 0.280	1.284	0.973	1.75*x + (-3.48)
	250	66.6 (52.49- 86.50)	504.317 (280.94- 1649.78)	1.458 ± 0.263	0.592	0.983	1.5*x + (-2.75)
	500	37.804 (28.86- 46.19)	191.416 (138.37–330.47)	1.819 ± 0.266	0.728	0.984	1.75*x + (-2.68)
	1000	23.934 (17.89- 29.31)	82.941 (67.89- 110.45)	2.374 ± 0.31	0.016	1	2.50*x + (-3.25)
*Malathion (P.C.)	125	51.105 (42.490- 60.291)	204.88 (153.02- 324.89)	2.125 ± 0.273	0.418	0.994	2.50*x + (-4.25)
	250	28.624 (20.152- 36.077)	149.309 (110.52- 247.82)	1.787 ± 0.273	0.156	0.996	1.75*x + (-2.48)
	500	18.823 (11.220- 25.404)	98.327 (75.63- 150.91)	1.785 ± 0.294	0.001	1	1.88*x + (-2.44)
	1000	18.018 (12.952- 22.176)	48.661 (40.88- 62.15)	2.97 ± 0.451	1.219	1	2.50*x + -(3.00)

*Significant Pearson goodness-of-fit test chi-square X^2^ ≥ 0.05 for all probit models *R^2^ regression coefficient *Y Equation regression, *P.C. positive control.

### Effects of titanium oxide nanoparticles on the total protein concentration of *I. aegyptiaca*

**Fig. 11:**
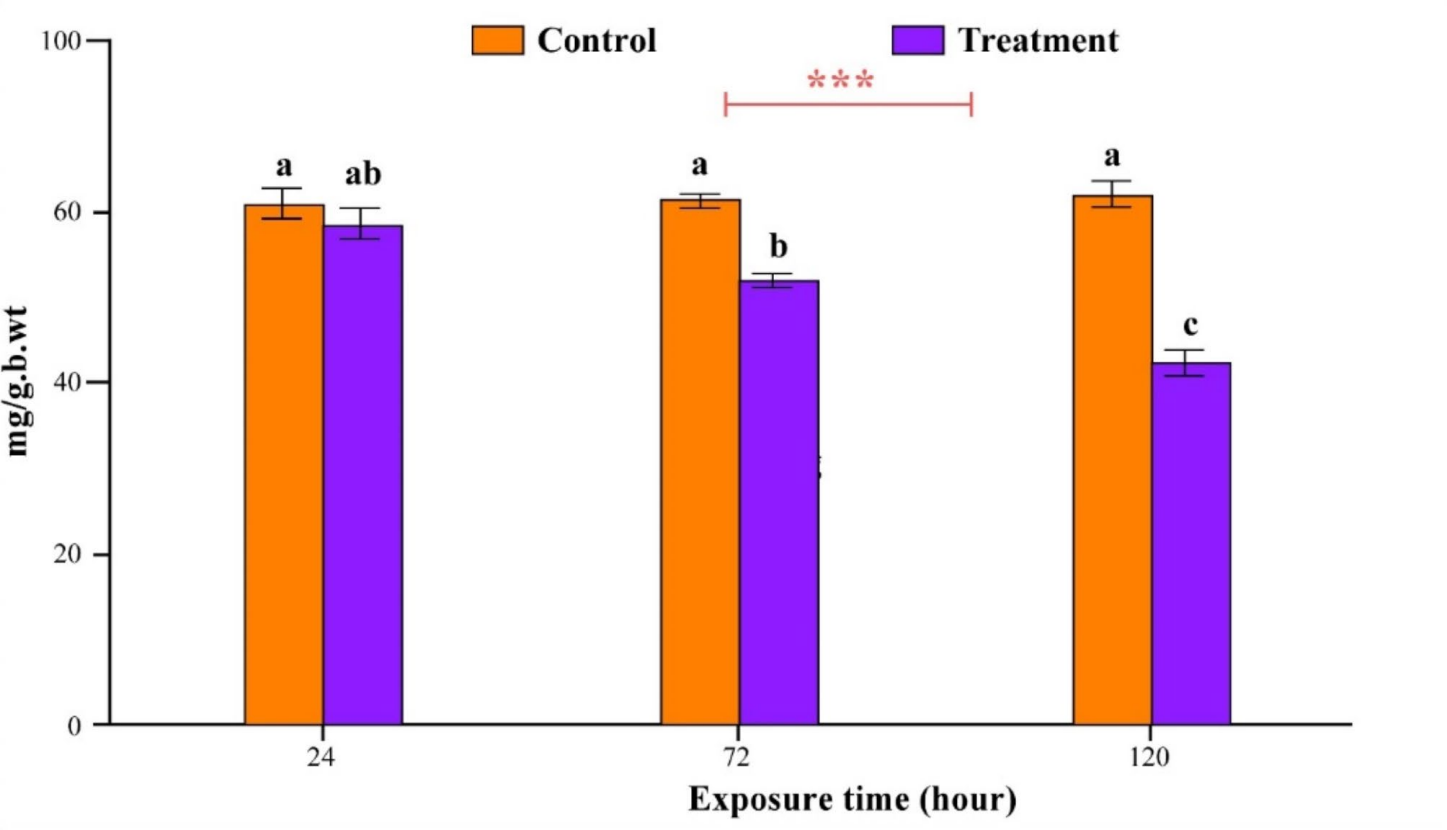
Total protein content of *Icerya aegyptiaca* treated with (124.112 ppm) TiO_2_-NPs after 24, 72 and 120 h of treatment. F _(5,12)_ value = 24.98*** at p = 0.00. Different letters indicate significance according to the Tukeyʼ HSD test at p ≤ 0.05 L.S.R ± SE = 7.41 ± 2.21 *** indicates significance at the 1% level for the treatment times and tested concentrations.

The total protein of *I. aegyptiaca* treated with (124.112 ppm) TiO_2_-NPs was illustrated in Fig. 11. One-way ANOVA revealed that, compared with the control (61.47 ± 1.73 mg/g.b.wt), the treatments induced not significantly increase (58.77 ± 1.97 mg/g.b.wt) in the total protein content of the insects after 24 h of exposure, followed by a significant reduction after 72 and 120 h of treatment (52.17 ± 1.48 and 42.63 ± 1.58 mg/g.b.wt), respectively, compared with the control (61.77 ± 0.91 and 62.47 ± 1.51 mg/g.b.wt), respectively.

### Protein profiles by polyacrylamide gel electrophoresis

**Fig. 12:**
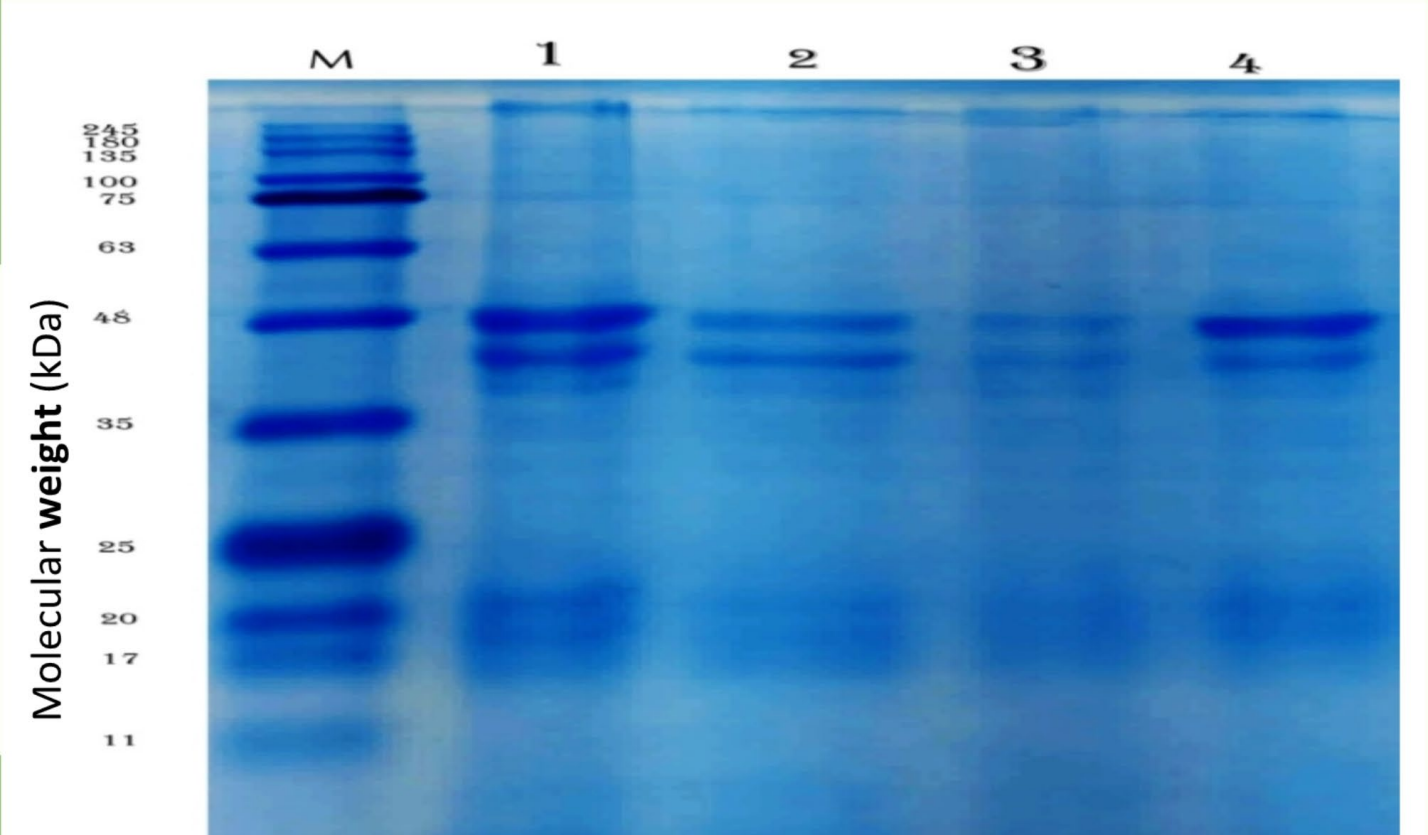
Photograph of electrophoretic protein patterns of *I. aegyptiaca* treated with (124.112 ppm) TiO_2_-NPs after 24, 72 and 120 h of treatment. M: Marker; 1: control sample; 2: treated after 24 h; 3: treated samples after 72 h; and 4: treated samples after 120 h**.**

The proteins from normal and treated insects treated with (124.112 ppm) TiO_2_-NPs were separated via SDS‒PAGE. The molecular weights of the proteins separated from normal and treated insects can be determined by comparing their electrophoretic mobility with that of known protein markers (Fig. 12). There was an increase in the intensity of the peptide bands corresponding to 48 and 43 kDa in the normal insects as well as in the treated insects on the fifth day after treatment in comparison with the samples from the first and third days of treatment. The protein bands of the normal and treated insects are compared in Table 7 according to their molecular weight and relative fragmentation (Rf), which represent the relative mobility of the protein bands. The results revealed that bands 7, 8 and 14 were common between the control sample and treated samples, with molecular weights of 48, 43 and 17 kDa and Rf values of 0.306, 0.362 and 0.783, respectively. Bands 12 and 15 appeared only in the control sample and disappeared in the treated samples, where their weights were 21 and 16 kDa and Rf values of 0.712 and 0.782, respectively. For the treated sample on the third day of exposure, a unique band with a molecular weight of 39 kDa and Rf of 0.395 appeared, but this band did not appear in the control sample or the other treated samples.

**Table 7:** Molecular weight (kDa) and relative fragmentation of *I. aegyptiaca* treated with (124.112 ppm) TiO_2_-NPs after 24, 72 and 120 h of treatment. M: Marker; 1: control sample; 2: treated sample after 24 h; 2: treated samples after 72 h; and 4: treated samples after 120 h.

Lanes	Parameters		1		2		3		4	
	Marker		Lane 1		Lane 2		Lane 3		Lane 4	
Raws	Mw*	Rf*	Mw	Rf	Mw	Rf	Mw	Rf	Mw	Rf
1	245	0.028								
2	180	0.046								
3	135	0.063								
4	100	0.103								
5	75	0.132								
6	63	0.205								
7	48	0.306	48	0.306	48	0.306	48	0.306	48	0.306
8			43	0.362	43	0.362	43	0.362	43	0.362
9							39	0.395		
10	35	0.452								
11	25	0.625								
12			21	0.712						
13	20	0.730			20	0.730	20	0.730	20	0.730
14	17	0.783	17	0.783	17	0.783	17	0.783	17	0.783
15			16	0.789						
16	11	0.901								
Bands no	12		6		4		5		4	

*RF (Relative Fragmentation) *Mw (Molecular weight).

## Discussion

Marine actinomycetes, especially those from sediment, are considered useful sources of unique and promising compounds because of the presence of a many biosynthesis-related gene clusters that increase their capacity to create many bioactive chemicals^38,39^. In the present investigation, actinomycete isolated from marine sediment were identified morphologically and confirmed via molecular methods, such as *Streptomyces rubrolavendulae,* given the code MNC2. The synthesis of nanoparticles uses Streptomyces, where microorganisms reduce or cap agents according to their catabolism in the media and generate the oxidation‒reduction process, leading to nanoparticle synthesis ^40^.

The MNCs produced TiO_2_-NPs in the present study were observed as coalescent white clusters and confirmed by UV screening with a visible wavelength of 335.52 nm. Additionally, the FTIR spectrum of the TiO_2_-NPs (3242 and 1625 cm^-1^) agreed with earlier reports by Chougala, et al. ^41^ and Panda, et al. ^42^.

SEM images revealed that the TiO_2_ nanoparticle beads were agglomerated and spherical, whereas EDX analysis revealed that the elemental composition of the TiO_2_ nanoparticles was composed of oxygen (47.52 and 73.04% atomic weight) and titanium (52.48 and 26.96% atomic weight). These results agreed with earlier reports by Islam et al. and Nino-Martinez et al. ^43,44^. The resulting diffraction peaks of TiO_2_-NPs from the XRD analysis were observed at 2θ values and were in good agreement with the characteristic peaks of TiO_2_ nanoparticles (Ref. Code: 01–075-2547) ^45^. TEM analysis of the studied nanoparticles revealed that the average grain size ranged from 21.5–28.7 nano meters, which was also confirmed by dynamic light scattering analysis and was in agreement with previous reports Panda*, *et al*.* ^42^.

Accordingly, the mortality percentage and probit analysis results of the bio-TiO_2_-NPs revealed that the bio-TiO_2_-NPs had good toxicological effects on *I. aegyptiaca* females. Our results are in agreement with those of Gutiérrez-Ramírez, et al. ^46^ and Shyam-Sundar, et al. ^47^, who reported that titanium oxide is promising for the control of *Bactericeracockerelli*, *A. aegypti* and *S. litura*. Compared with those of the control, the quantitative and qualitative total protein contents of the *I. aegyptiaca* exposed to the LC_50_ of TiO_2_-NPs for 24, 72, and 120 h sharply increased, and the total protein content of the insects after 24 h of exposure significantly decreased after 72 and 120 h of treatment. Several writers have reported that metal nanoparticles deplete protein levels in different insects. Osman, et al. ^48^ and Fouad, et al. ^49^ revealed that silica, zinc and silver nanoparticles caused decreases in total carbohydrate and total protein contents in treated *S. littoralis* and *Aedes albopictus* larvae. Kos, et al. ^50^ reported a reduction in the protein levels of different body organs in honeybees (*Apis mellifera*) when treated with cerium oxide nanoparticles.

As confirmed by electrophoretic analysis via SDS‒PAGE, compared with the control, TiO_2_-NP spraying caused fluctuations in the protein patterns of *Icerya aegyptiaca,* such as the disappearance of normal bands and the appearance of abnormal bands. These findings were in line with those of Hassan, et al. ^27^, who detected differences in the protein patterns of untreated and treated *S. littoralis* larvae with indoxacarb, spinetoram, and methoxyfenozide. Radwan and Taha ^51^ reported a correlation between the loss of various proteins and the reduction in band intensity when SDS‒PAGE was used. In addition, Farag, et al.^52^ reported the disappearance of normal protein bands in *Culex pipiens* larvae treated with pomegranate peel extracts. . The observed depletion of protein may be due to the increased need for energy to overcome the stress caused by the nanoparticles, which may have resulted from the protein breaking down into amino acids. In nanomaterial-stressed organisms, a mechanism for meeting energy needs might be affected by the depletion of proteins and other nutrients caused by nanomaterials ^53^.

The contact of TiO_2_-NPs with *I. aegyptiaca* occurs in two ways. The first is the adhesion of the nanoparticles on the outer body surface, which could change the properties of the membrane and affect permeability and cell respiration ^54,55^. The second route involves nano-infestation, which is absorbed via the gastrointestinal tract and distributed throughout different organs in the body. There is a direct proportional relationship between TiO_2_-NP toxicity and their size or physicochemical properties, where an overdose of smaller nanoparticles with a large surface area and hydrodynamic diameter results in greater toxicity ^56,57^. Insects exposed to nanoparticles present increased levels of reactive oxygen species, which are among the most harmful cellular effects. Stanić and Tanasković ^58^ regarding the antibacterial effect of TiO_2_-NPs on their ability to initiate several oxidation processes through the generation of reactive oxidative stress that causes damage to the cell outer membrane ^59^, which may be due to the negative charge carried by the TiO_2_-NP surface, as confirmed by the zeta potential test. Tuncsoy and Mese ^60^ reported that TiO_2_-NPs cause severe oxidative stress to *Galleria mellonella larvae* by disturbing several antioxidant enzymes, such as catalase and reduced glutathione. Moreover, Zorlu, et al. ^61^ reported a decrease in protein synthesis in a *Galleria mellonella* insect model treated with a high dose of titanium nanoparticles, which may be attributed in part to the increased use of some proteins, such as metal-binding proteins, heat shock proteins (HSPs), and metallothionein (MTs), to block nanoparticle toxicity and to the increased use of lipoproteins to prevent damage to the antioxidant system. According to the present TiO_2_-NP Zeta potential test, the surface negative charges of the nanoparticles give them the ability to attract and combine with calcium in the insect body. Cameron, et al. ^62^ reported that titanium could affect the intracellular calcium level, which in turn altered the synthesis of Ca^2+^-binding proteins. Additionally, another scenario discussed by Lee, et al. ^63^ is that the increase in calcium in its free form causes a response to cellular stress that may promote autophagy, which damages proteins and organelles ^64^. According to Assar, et al. ^65^, protein loss occurs during intoxication as a result of reduced body weight, protein breakdown to release energy, or a substance’s direct impact on the transport of amino acids by cells.

By comparing the effects of TiO2-NPs with those of malathion on cultivated crops or plant activity, Rodríguez-González, et al. ^66^ demonstrated the advantages of the use of titanium oxide or titanium in their new forms on cultivated plants and reported that a progressive elongation in roots helps in plant growth regulation, increases plant photosynthesis and enhances germination; moreover, TiO-NPs can detoxify any excess pesticide or metal toxins in the soil ^67,68^. On the other hand, malathion has a reduction effect on nitrogen-fixing soil microbes in the soil and reduces plant enzyme activity, which may lead to a decrease in plant or crop activity and a decrease in seed production^69^.

## Conclusion

From the present study and the other study, it can be concluded that

1. The green synthesis of nanoparticles via microorganisms is an effective method because of its low cost, environmental friendliness, and ability to be used in many applications, such as agriculture and medicine.
2. Actinomycetes have a high capacity for titanium nanoparticle synthesis.
3. The TiO_2_-NPs were confirmed by X-ray diffraction (XRD), Fourier transform infrared (FTIR), ultraviolet (UV), transmission electron microscopy (TEM), scanning electron microscopy (SEM), zeta potential, and scanning electron microscopy (SEM), which revealed that the average size was 21.5–28.7 nm, with a negative surface charge.
4. Even the insecticidal effect of TiO_2_-NPs against female *Icerya aegyptiaca* resulted in good LC_50 & 90_ and LT_50 & 90_ values compared with recommended insecticide (malathion),.
5. The total protein level in female *Icerya aegyptiaca* significantly decreased as a result of the insecticidal effect of TiO_2-_NPs, with the disappearance of most of the protein bands detected via SDS‒PAGE for female* Icerya aegyptiaca*
6. The insecticidal effect of TiO_2_-NPs may be attributed to their ability to propagate an oxidative effect or their ability to attract calcium, which has a role in protein synthesis or decreases the synthesis of some enzymatic antioxidants.

## Electronic supplementary material

Below is the link to the electronic supplementary material.

Supplementary Material 1

Supplementary Material 2

Supplementary Material 3

Supplementary Material 4

Supplementary Material 5

Supplementary Material 6

Supplementary Material 7

Supplementary Material 8

## Data Availability

The authors submitted the sequence of the isolate, which is 99.9% like Streptomyces rubrolavendulae, according to the Nucleotides alignment to the GenBank1 Nucleotide Sequence Database, and the following GenBank accession number(s) for the nucleotide sequence(s): SUB14289766 Streptomyces PP436747.
