# Supplementary material for: Biogenic synthesis of titanium nanoparticles by Streptomyces rubrolavendulae for sustainable management of Icerya aegyptiaca (Douglas)
Source: Sci Rep. 2025 Jan 9;15:1380. doi: 10.1038/s41598-024-81291-4 (PMC11711640; doi:10.1038/s41598-024-81291-4)

Zeta Potential Report
v2.2

Malvern Instruments Ltd - © Copyright 2008

Sample Details

Sample Name: 1 1

SOP Name: mansettings.nano

General Notes: This SOP is also suitable for most samples of conductivity less than 5 mS.

**File Name:**	Dr. Inas Abou Elenain.dts	**Dispersant Name:**	Water
**Record Number:**	4	**Dispersant RI:**	1.330
**Date and Time:**	Sunday, February 18, 2024 8:46:...	**Viscosity (cP):**	0.8872
		**Dispersant Dielectric Constant:**	78.5

System

**Temperature (°C):**	24.9	**Zeta Runs:**	12
**Count Rate (kcps):**	259.9	**Measurement Position (mm):**	2.00
**Cell Description:**	Clear disposable zeta cell	**Attenuator:**	7

Results

			**Mean (mV)**	**Area (%)**	**Width (mV)**
**Zeta Potential (mV):**	-29.6	**Peak 1:**	-29.6	100.0	5.61
**Zeta Deviation (mV):**	5.61	**Peak 2:**	0.00	0.0	0.00
**Conductivity (mS/cm):**	0.0124	**Peak 3:**	0.00	0.0	0.00

Result quality : Good

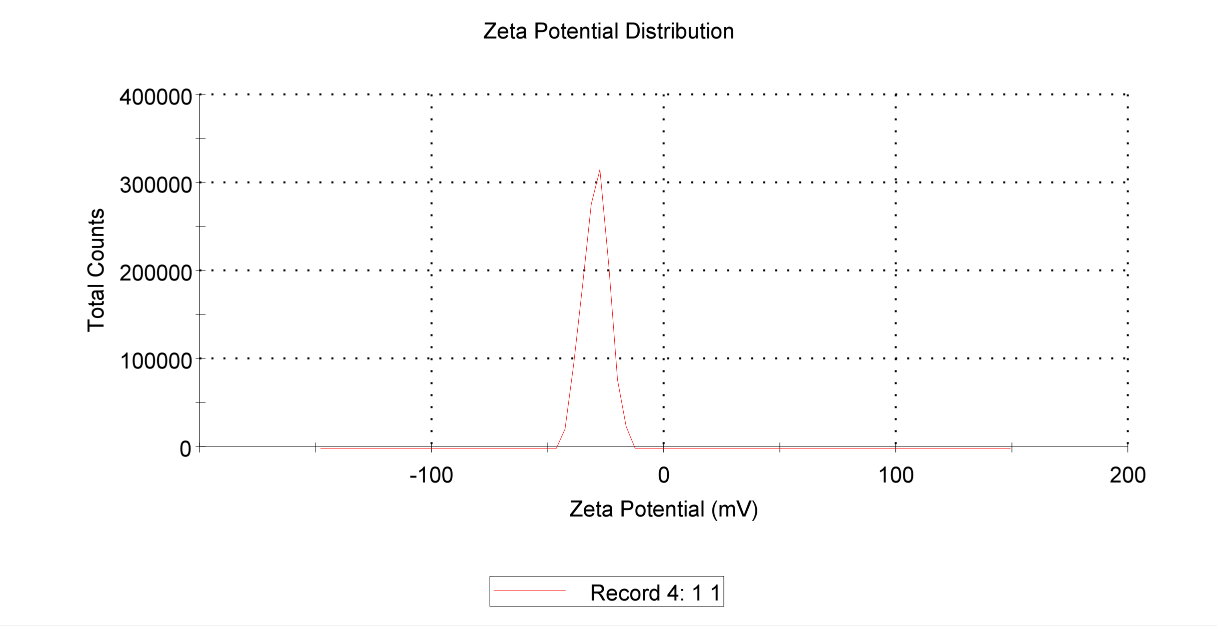

Supplement: Supplementary file 2 — Supplementary Material 2 [file 41598_2024_81291_MOESM2_ESM.pdf]
