# Supplementary material for: Biogenic synthesis of titanium nanoparticles by Streptomyces rubrolavendulae for sustainable management of Icerya aegyptiaca (Douglas)
Source: Sci Rep. 2025 Jan 9;15:1380. doi: 10.1038/s41598-024-81291-4 (PMC11711640; doi:10.1038/s41598-024-81291-4)

Size Distribution Report by Intensity
v2.1

Sample Details

**Sample Name:**	1 1
**SOP Name:**	mansettings.nano
**General Notes:**	This SOP is also suitable for most samples of conductivity less than 5 mS.

**File Name:**	Dr. Inas Abou Elenain.dts	**Dispersant Name:**	Water
**Record Number:**	1	**Dispersant RI:**	1.330
**Material RI:**	1.59	**Viscosity (cP):**	0.8872
**Material Absorbtion:**	0.010	**Measurement Date and Time:**	Sunday, February 18, 2024 8:...

System

**Temperature (°C):**	24.9	**Duration Used (s):**	60
**Count Rate (kcps):**	338.3	**Measurement Position (mm):**	5.50
**Cell Description:**	Clear disposable zeta cell	**Attenuator:**	7

Results

			Size (d.nm):	% Intensity	Width (d.nm):
**Z-Average (d.nm):**	885.1	**Peak 1:**	653.8	87.5	142.0
**PdI:**	0.597	**Peak 2:**	156.4	12.5	25.07
**Intercept:**	0.848	**Peak 3:**	0.000	0.0	0.000

Result quality : **Refer to quality report**

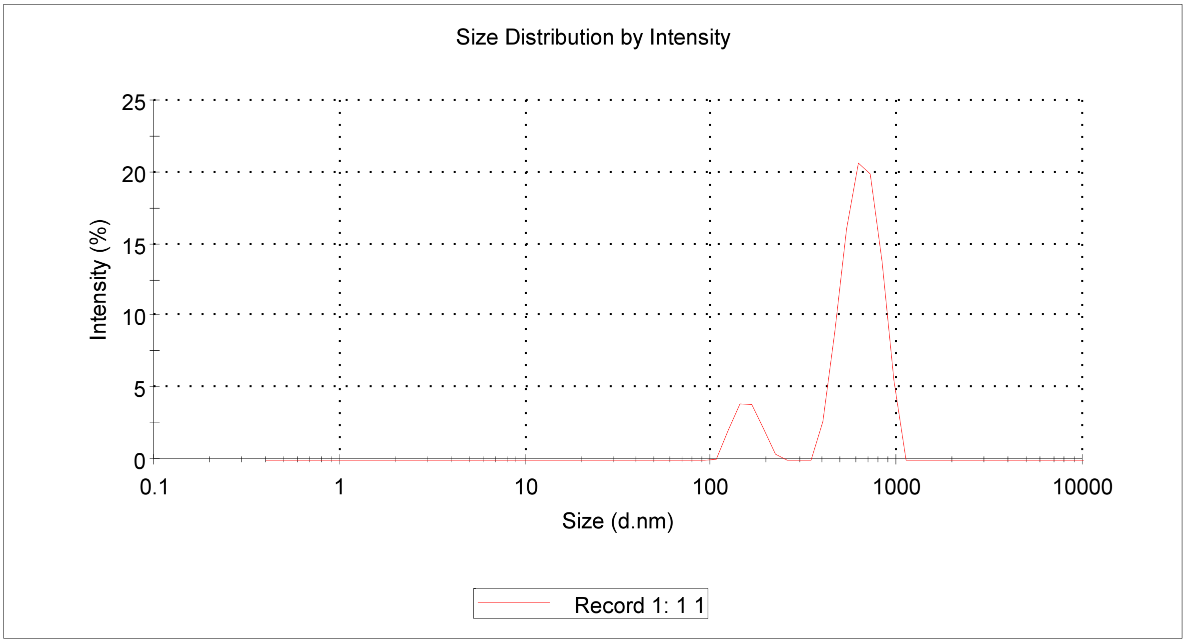

Supplement: Supplementary file 3 — Supplementary Material 3 [file 41598_2024_81291_MOESM3_ESM.pdf]
