# Supplementary material for: Biogenic synthesis of titanium nanoparticles by Streptomyces rubrolavendulae for sustainable management of Icerya aegyptiaca (Douglas)
Source: Sci Rep. 2025 Jan 9;15:1380. doi: 10.1038/s41598-024-81291-4 (PMC11711640; doi:10.1038/s41598-024-81291-4)

Size Statistics Report by Number
v2.0

Malvern

Malvern Instruments Ltd - © Copyright 2008

Sample Details

Sample Name: 1 1

File Name: Dr. Inas Abou Elenain.dts

SOP Name: mansettings.nano

Measurement Date and Time: Sunday, February 18, 2024 8:41:23 AM

**Z-Average (nm):**	885.1331	**Derived Count Rate (kcps):**	26821.8266215...
**Standard Deviation:**	0	**Standard Deviation:**	0
**%Std Deviation:**	0	**%Std Deviation:**	0
**Variance:**	0	**Variance:**	0

Size d.nm	Mean Number %	Std Dev Number %	Size d.nm	Mean Number %	Std Dev Number %	Size d.nm	Mean Number %	Std Dev Number %	Size d.nm	Mean Number %	Std Dev Number %
0.4000	0.0		5.615	0.0		78.82	0.0		1106	0.2	
0.4632	0.0		6.503	0.0		91.28	0.5		1281	0.0	
0.5365	0.0		7.531	0.0		105.7	8.4		1484	0.0	
0.6213	0.0		8.721	0.0		122.4	22.4		1718	0.0	
0.7195	0.0		10.10	0.0		141.8	25.9		1990	0.0	
0.8332	0.0		11.70	0.0		164.2	17.1		2305	0.0	
0.9649	0.0		13.54	0.0		190.1	7.6		2669	0.0	
1.117	0.0		15.69	0.0		220.2	2.0		3091	0.0	
1.294	0.0		18.17	0.0		255.0	0.2		3580	0.0	
1.499	0.0		21.04	0.0		295.3	0.0		4145	0.0	
1.736	0.0		24.36	0.0		342.0	0.3		4801	0.0	
2.010	0.0		28.21	0.0		396.1	1.1		5560	0.0	
2.328	0.0		32.67	0.0		458.7	2.3		6439	0.0	
2.696	0.0		37.84	0.0		531.2	3.1		7456	0.0	
3.122	0.0		43.82	0.0		615.1	3.3		8635	0.0	
3.615	0.0		50.75	0.0		712.4	2.9		1.000e4	0.0	
4.187	0.0		58.77	0.0		825.0	1.9				
4.849	0.0		68.06	0.0		955.4	0.9				

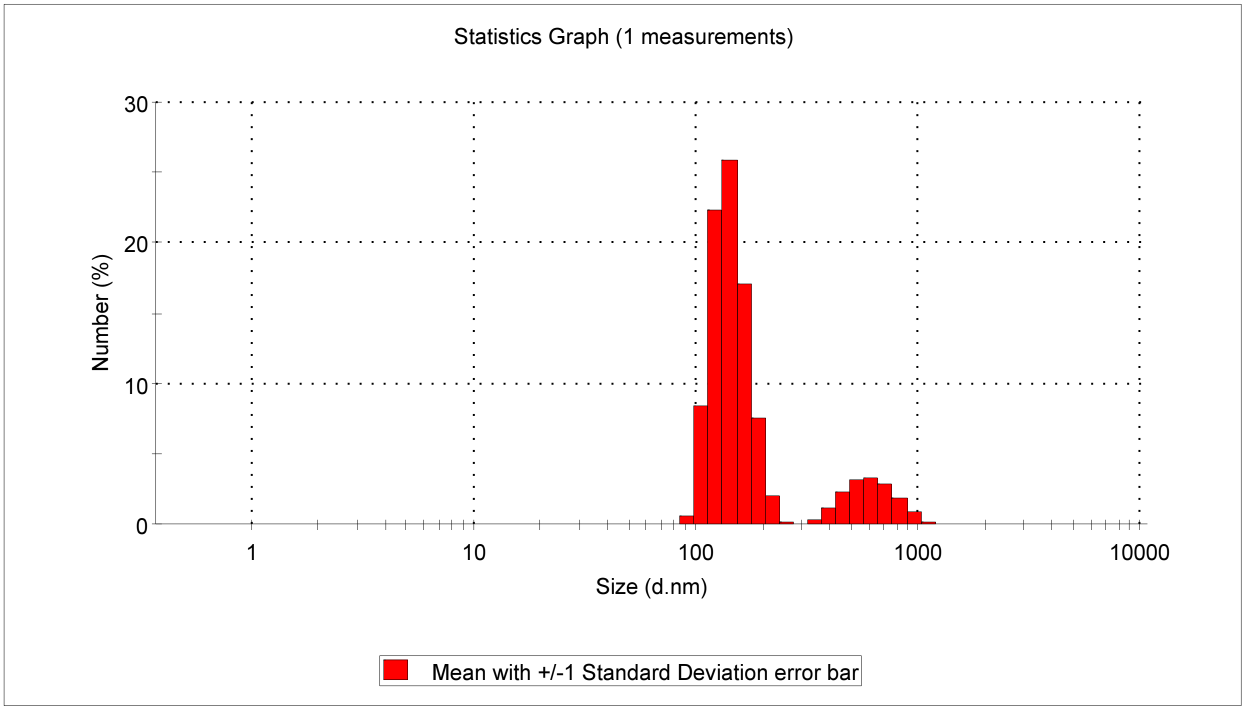

Supplement: Supplementary file 4 — Supplementary Material 4 [file 41598_2024_81291_MOESM4_ESM.pdf]
