# Supplementary material for: Biogenic synthesis of titanium nanoparticles by Streptomyces rubrolavendulae for sustainable management of Icerya aegyptiaca (Douglas)
Source: Sci Rep. 2025 Jan 9;15:1380. doi: 10.1038/s41598-024-81291-4 (PMC11711640; doi:10.1038/s41598-024-81291-4)

Size Distribution Report by Number

v2.1

Sample Details

Sample Name: 1 1

SOP Name: mansettings.nano

General Notes: This SOP is also suitable for most samples of conductivity less than 5 mS.

**File Name:**	Dr. Inas Abou Elenain.dts	**Dispersant Name:**	Water
**Record Number:**	1	**Dispersant RI:**	1.330
**Material RI:**	1.59	**Viscosity (cP):**	0.8872
**Material Absorbtion:**	0.010	**Measurement Date and Time:**	Sunday, February 18, 2024 8...

System

**Temperature (°C):**	24.9	**Duration Used (s):**	60
**Count Rate (kcps):**	338.3	**Measurement Position (mm):**	5.50
**Cell Description:**	Clear disposable zeta cell	**Attenuator:**	7

Results

			Size (d.nm):	% Number	Width (d.nm...
**Z-Average (d.nm):**	885.1	**Peak 1:**	621.6	16.0	159.3
**PdI:**	0.597	**Peak 2:**	143.8	84.0	27.04
**Intercept:**	0.848	**Peak 3:**	0.000	0.0	0.000

Result quality : **Refer to quality report**

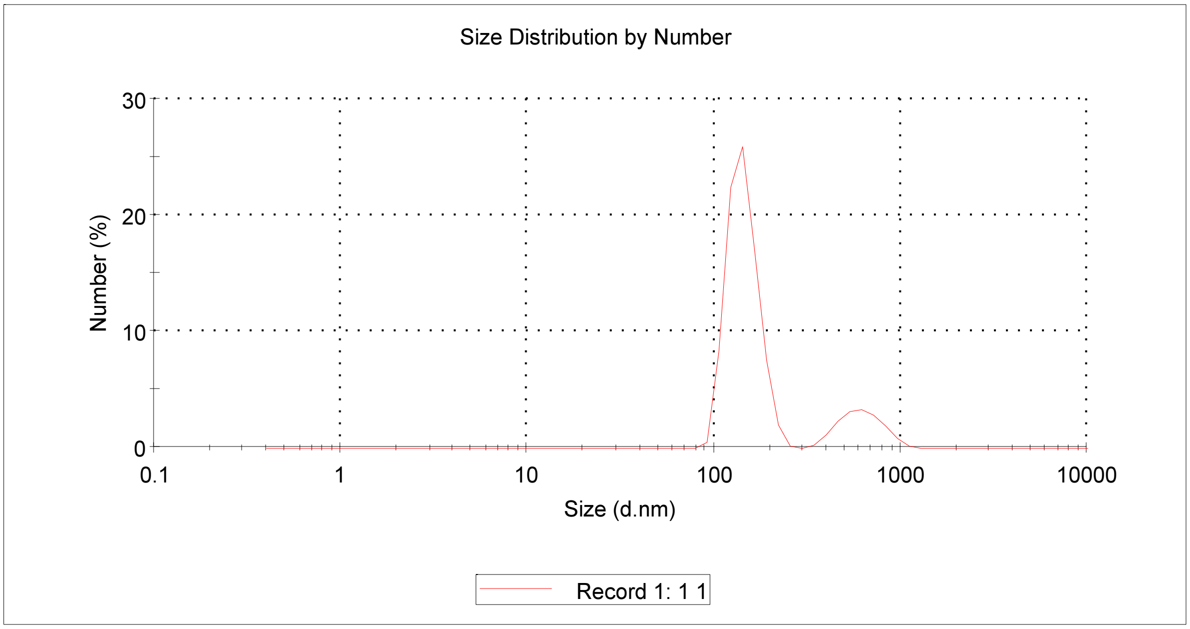

Supplement: Supplementary file 5 — Supplementary Material 5 [file 41598_2024_81291_MOESM5_ESM.pdf]
