# Supplementary material for: Biogenic synthesis of titanium nanoparticles by Streptomyces rubrolavendulae for sustainable management of Icerya aegyptiaca (Douglas)
Source: Sci Rep. 2025 Jan 9;15:1380. doi: 10.1038/s41598-024-81291-4 (PMC11711640; doi:10.1038/s41598-024-81291-4)
Supplement: Supplementary file 6 — Supplementary Material 6 [file 41598_2024_81291_MOESM6_ESM.pdf]

Sample ID:IR-2024-1

Method Name:C:\Users\Public\Documents\Agilent\MicroLab\Methods\A-Transmission.a2m

Sample Scans:16

User:Administrator

Background Scans:16

Date/Time:02/14/2024 12:44:55 PM

Resolution:16

Range:4000 - 650

System Status:Good

Apodization:Happ-Genzel

File Location:C:\Users\Public\Documents\Agilent\MicroLab\Results\\IR-2024-1_2024-02-14T12-44-55.a2r

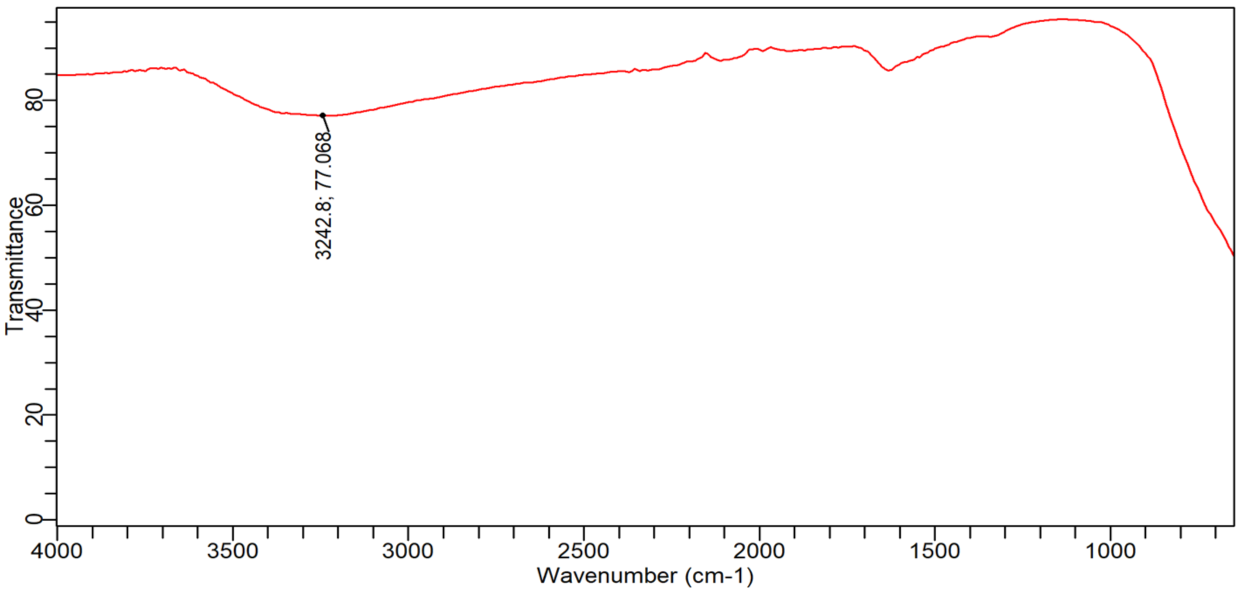

Peak Number	Wavenumber (cm^{-1})	Intensity
1	3242.78392	77.06823
