# Supplementary material for: Biogenic synthesis of titanium nanoparticles by Streptomyces rubrolavendulae for sustainable management of Icerya aegyptiaca (Douglas)
Source: Sci Rep. 2025 Jan 9;15:1380. doi: 10.1038/s41598-024-81291-4 (PMC11711640; doi:10.1038/s41598-024-81291-4)

Name and formula

Reference code:	01-075-2547
Mineral name:	Anatase, syn
Compound name:	Titanium Oxide
PDF index name:	Titanium Oxide
Empirical formula:	O_2Ti
Chemical formula:	TiO_2

Crystallographic parameters

Crystal system:	Tetragonal
Space group:	I41/amd
Space group number:	141
a (Å):	3.7860
b (Å):	3.7860
c (Å):	9.4950
Alpha (°):	90.0000
Beta (°):	90.0000
Gamma (°):	90.0000
Volume of cell (10^6 pm^3):	136.10
Z:	4.00
RIR:	5.03

Status, subfiles and quality

Status:	Alternate Pattern
Subfiles:	Alloy, metal or intermetalic
	Common Phase
	Excipient
	Forensic
	ICSD Pattern
	Inorganic
	Mineral
	Pharmaceutical
Quality:	Star (S)

Comments

ANX:	AX2
ICSD collection code:	154604
Creation Date:	9/1/2008
Modification Date:	9/1/2011
Cross-References:	ICDD:01-075-2547, ICSD:154604
ANX:	AX2
Analysis:	O2 Ti1
Formula from original source:	Ti O2
ICSD Collection Code:	154604
Calculated Pattern Original Remarks:	Annealed Fe doped sample
Wyckoff Sequence:	e b(I41/AMDZ)
Unit Cell Data Source:	Powder Diffraction.

References

Primary reference: Djerdj, I., Tonejc, A.M., J. Alloys Compd., **413**, 159, (2006)
Structure: Djerdj, I., Tonejc, A.M., J. Alloys Compd., **413**, 159, (2006)

Peak list

No.	h	k	l	d [Å]	2Theta[deg]	I [%]
1	1	0	1	3.51674	25.305	100.0
2	1	0	3	2.42826	36.990	4.9
3	0	0	4	2.37375	37.871	16.8
4	1	1	2	2.33191	38.578	7.1
5	2	0	0	1.89300	48.023	24.4
6	1	0	5	1.69744	53.976	15.0
7	2	1	1	1.66686	55.049	14.7
8	2	1	3	1.49295	62.123	1.9
9	2	0	4	1.48001	62.727	9.5
10	1	1	6	1.36229	68.866	5.0
11	2	2	0	1.33855	70.265	4.8
12	1	0	7	1.27695	74.204	0.4
13	2	1	5	1.26377	75.110	7.2
14	3	0	1	1.25100	76.012	1.9
15	0	0	8	1.18687	80.935	0.2
16	3	0	3	1.17225	82.160	0.3
17	2	2	4	1.16595	82.701	3.0
18	3	1	2	1.16089	83.141	1.1
19	2	1	7	1.05861	93.380	0.3
20	3	0	5	1.05107	94.255	1.5
21	3	2	1	1.04368	95.133	1.6
22	1	0	9	1.01628	98.569	0.9
23	2	0	8	1.00557	99.998	0.4
24	3	2	3	0.99663	101.231	0.3
25	3	1	6	0.95478	107.565	2.1
26	4	0	0	0.94650	108.946	1.0
27	3	0	7	0.92395	112.962	0.1
28	3	2	5	0.91892	113.914	1.9
29	4	1	1	0.91398	114.874	1.0
30	2	1	9	0.89488	118.809	2.0
31	1	1	10	0.89488	118.809	2.0
32	2	2	8	0.88805	120.316	0.3
33	4	1	3	0.88187	121.731	0.3
34	4	0	4	0.87919	122.363	1.0
35	3	3	2	0.87701	122.882	0.2
36	4	2	0	0.84658	130.983	1.5
37	1	0	11	0.84159	132.495	0.3
38	4	2	2	0.83343	135.112	0.1
39	3	2	7	0.83032	136.161	0.2
40	4	1	5	0.82667	137.437	1.5
41	3	0	9	0.80942	144.227	0.5

Structure

No.	Name	Elem.	X	Y	Z	Biso	sof	Wyck.
1	TI1	Ti	0.00000	0.25000	0.37500	1.2000	1.0000	4b
2	O1	O	0.00000	0.25000	0.17130	0.8000	1.0000	8e

Stick Pattern

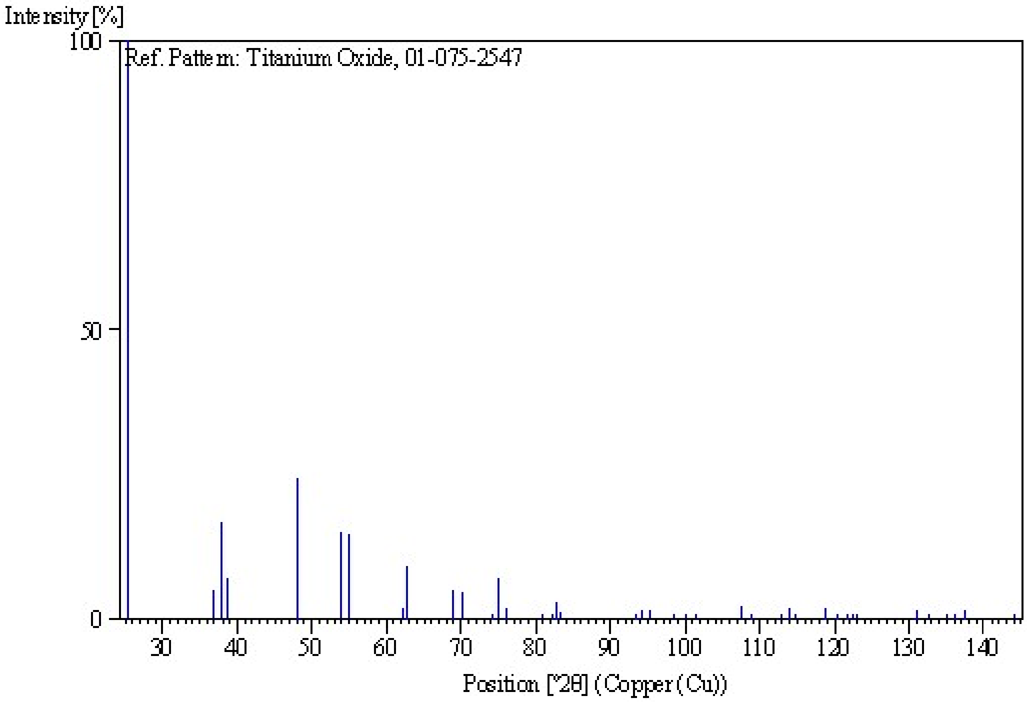

Supplement: Supplementary file 8 — Supplementary Material 8 [file 41598_2024_81291_MOESM8_ESM.pdf]
